# GPR101 mediates the pro-resolving actions of RvD5_n-3 DPA_ in arthritis and infections

**DOI:** 10.1172/JCI131609

**Published:** 2019-12-03

**Authors:** Magdalena B. Flak, Duco S. Koenis, Agua Sobrino, James Smith, Kimberly Pistorius, Francesco Palmas, Jesmond Dalli

**Affiliations:** 1 William Harvey Research Institute, Barts and The London School of Medicine and Dentistry, and; 2 Centre for Inflammation and Therapeutic Innovation, Queen Mary University of London, Charterhouse Square, London, United Kingdom.

**Keywords:** Infectious disease, Inflammation, Arthritis, Bacterial infections, G protein&ndash;coupled receptors

## Abstract

N-3 docosapentaenoic acid–derived resolvin D5 (RvD5_n-3__DPA_) is diurnally regulated in peripheral blood and exerts tissue-protective actions during inflammatory arthritis. Here, using an orphan GPCR screening approach coupled with functional readouts, we investigated the receptor(s) involved in mediating the leukocyte-directed actions of RvD5_n-3 DPA_ and identified GPR101 as the top candidate. RvD5_n-3__DPA_ bound to GPR101 with high selectivity and stereospecificity, as demonstrated by a calculated *K_D_* of approximately 6.9 nM. In macrophages, *GPR101* knockdown limited the ability of RvD5_n-3__DPA_ to upregulate cyclic adenosine monophosphate, phagocytosis of bacteria, and efferocytosis. Inhibition of this receptor in mouse and human leukocytes abrogated the pro-resolving actions of RvD5_n-3__DPA_, including the regulation of bacterial phagocytosis in neutrophils. Knockdown of the receptor in vivo reversed the protective actions of RvD5_n-3__DPA_ in limiting joint and gut inflammation during inflammatory arthritis. Administration of RvD5_n-3__DPA_ during *E. coli*–initiated inflammation regulated neutrophil trafficking to the site of inflammation, increased bacterial phagocytosis by neutrophils and macrophages, and accelerated the resolution of infectious inflammation. These in vivo protective actions of RvD5_n-3__DPA_ were limited when *Gpr101* was knocked down. Together, our findings demonstrate a fundamental role for GPR101 in mediating the leukocyte-directed actions of RvD5_n-3__DPA_.

## Introduction

Development of the inflammatory response was a fundamental step in the evolution of multicellular organisms, given that this mechanism is vital in warding off invading pathogens and in coordinating reparative and regenerative mechanisms (1, 2). Leukocytes play a central role in the clearance of pathogens, in which, for example, neutrophils and macrophages are tasked with the uptake and killing of bacteria (3, 4). These cells are also important in coordinating the termination of acute inflammation as well as initiating tissue repair and regeneration (4–6). Central to the protective actions exerted by leukocytes is a novel genus of mediators termed specialized pro-resolving mediators (SPMs) (4, 7, 8). These autacoids are produced through the enzymatic conversion of essential fatty acids, which yields molecules with a defined stereochemistry and potent biological properties (4, 7, 8).

Recent studies demonstrate that in addition to arachidonic acid, eicosapentaenoic acid, and docosahexaenoic acid (DHA), n-3 docosapentaenoic acid (n-3 DPA) is also a substrate for conversion to novel, structurally distinct bioactive mediators (9, 10). One of these novel mediators, n-3 DPA–derived resolvin D5 (RvD5_n-3__DPA_) (7S,17S-dihydroxy-8E,10Z,13Z,15E,19Z-docosapentaenoic acid), is diurnally regulated in human peripheral blood, and loss of its production, together with that of the related mediators RvD1_n-3__DPA_ (7S,8R,17S-trihydroxy-9E,11E,13Z,15E,19Z-docosapentaenoic acid) and RvD2_n-3__DPA_ (7S,16,17S-trihydroxy-8,10*Z*,12,14,19Z- docosapentaenoic acid), in patients with cardiovascular disease is linked with increased peripheral blood neutrophil, monocyte, and platelet activation (9). RvD5_n-3__DPA_ also exerts tissue-protective actions by regulating mouse leukocyte trafficking and responses in vivo, as well as neutrophil, monocyte, and macrophage responses in vitro (9). We recently uncovered a homeostatic role for RvD5_n-3__DPA_ in regulating intestinal epithelial barrier function, where decreased levels of this mediator during inflammatory arthritis are linked with increased intestinal barrier permeability and joint inflammation (11). Treatment of arthritic mice with RvD5_n-3__DPA_ restored barrier function, reduced leukocyte trafficking into the arthritic joints, and limited joint inflammation (11).

The biological actions of lipid mediators are stereospecific, suggesting that they activate cognate receptors to evoke downstream protective activities (12). Studies investigating the identity of these proteins demonstrate that lipid mediators, including the classic eicosanoids and SPMs, activate a class of receptors known as GPCRs (12, 13). In this context, the first pro-resolving mediator receptor identified was the lipoxin A_4_ receptor, also referred to as formyl peptide receptor 2 (ALX) or FPR2 (14). This receptor was later found to also mediate the biological actions of DHA-derived resolvin D1 (7S,8R,17S-trihydroxy-4Z,9E,11E,13Z,15E,19Z-docosahexaenoic acid) as well as the pro-resolving protein annexin A1 (15, 16). Recent studies have uncovered a role for the orphan receptors GPR32 and GPR18 in mediating the biological actions of pro-resolving mediators, with RvD1 and RvD3 (4S,11R,17S-trihydroxy-5Z,7E,9E,13Z,15E,19Z-docosahexaenoic acid) activating GPR32 (16, 17) and RvD2 activating GPR18 (18). Of note, the identity of the receptor mediating the biological actions of RvD5_n-3__DPA_ is not known.

In the present study, we used an integrated approach to establish the identity of the GPCR that mediates the pro-resolving actions of RvD5_n-3__DPA_ on leukocytes. Results from these experiments identified GPR101 as a candidate receptor for RvD5_n-3__DPA_. Flow cytometric analysis demonstrated that GPR101 was expressed on both human and mouse peripheral blood leukocytes. Using radiolabeled RvD5_n-3__DPA_, we observed specific binding of this mediator to GPR101 with an affinity that was within its bioactive range. Knockdown of *Gpr101* in mice led to an abrogation of the protective actions of RvD5_n-3__DPA_ in vivo by limiting its ability to regulate inflammatory arthritis and infectious inflammation.

## Results

### Identification of candidate receptors for RvD5_n-3__DPA_.

**Figure 1:**
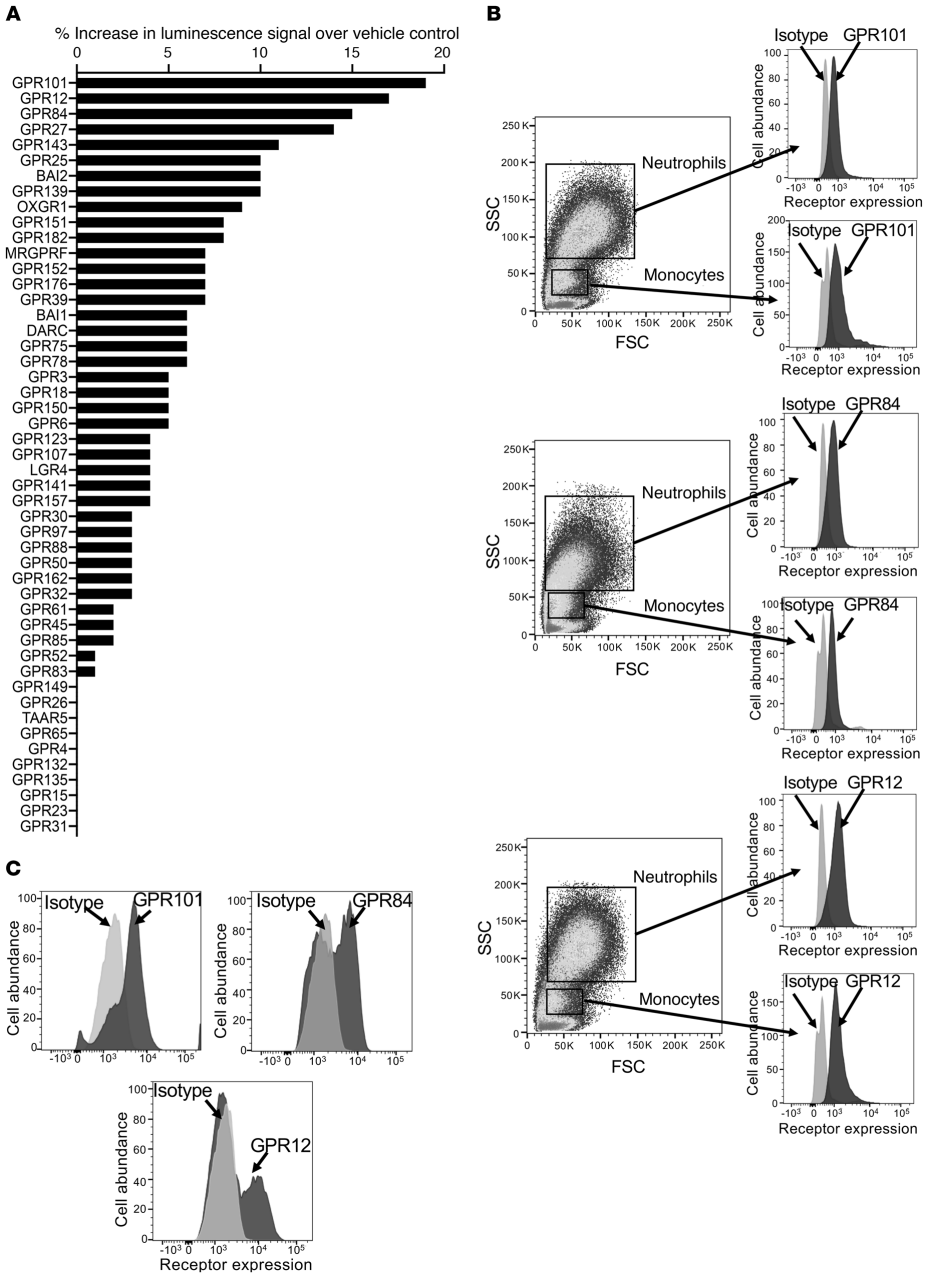
RvD5_n-3__DPA_ receptor candidates are expressed on human leukocytes. (**A**) Activation of orphan receptors by RvD5_n-3__DPA_ (10 nM). Results represent the percentage increase in luminescence signal over vehicle control. (**B** and **C**) Expression of the top 3 candidate receptors on human (**B**) peripheral blood leukocytes and (**C**) macrophages. Results are representative of 4 donors. FSC, forward scatter; SSC, side scatter.

In order to establish whether the biological actions of RvD5_n-3__DPA_ were mediated by a GPCR, we screened a panel of orphan GPCRs to determine whether RvD5_n-3__DPA_ showed agonistic activity toward these receptors, using 10 nM RvD5_n-3__DPA_ and assessing increases in luminescence as a readout for receptor activation. Here, we found that the strongest agonistic signals elicited by this pro-resolving mediator were with GPR101, GPR12, and GPR84 (Figure 1A), with values approximately 15%–20% above the control value. Given that RvD5_n-3__DPA_ regulates the biological actions of monocyte-derived macrophages and peripheral blood leukocytes (9, 10), we next investigated the expression of these 3 receptors on circulating human neutrophils and monocytes and identified all 3 receptors (Figure 1B). Moreover, human monocyte–derived macrophages also expressed all 3 receptors on their cell surface (Figure 1C).

### RvD5_n-3__DPA_ stereospecifically activates GPR101.

**Figure 2:**
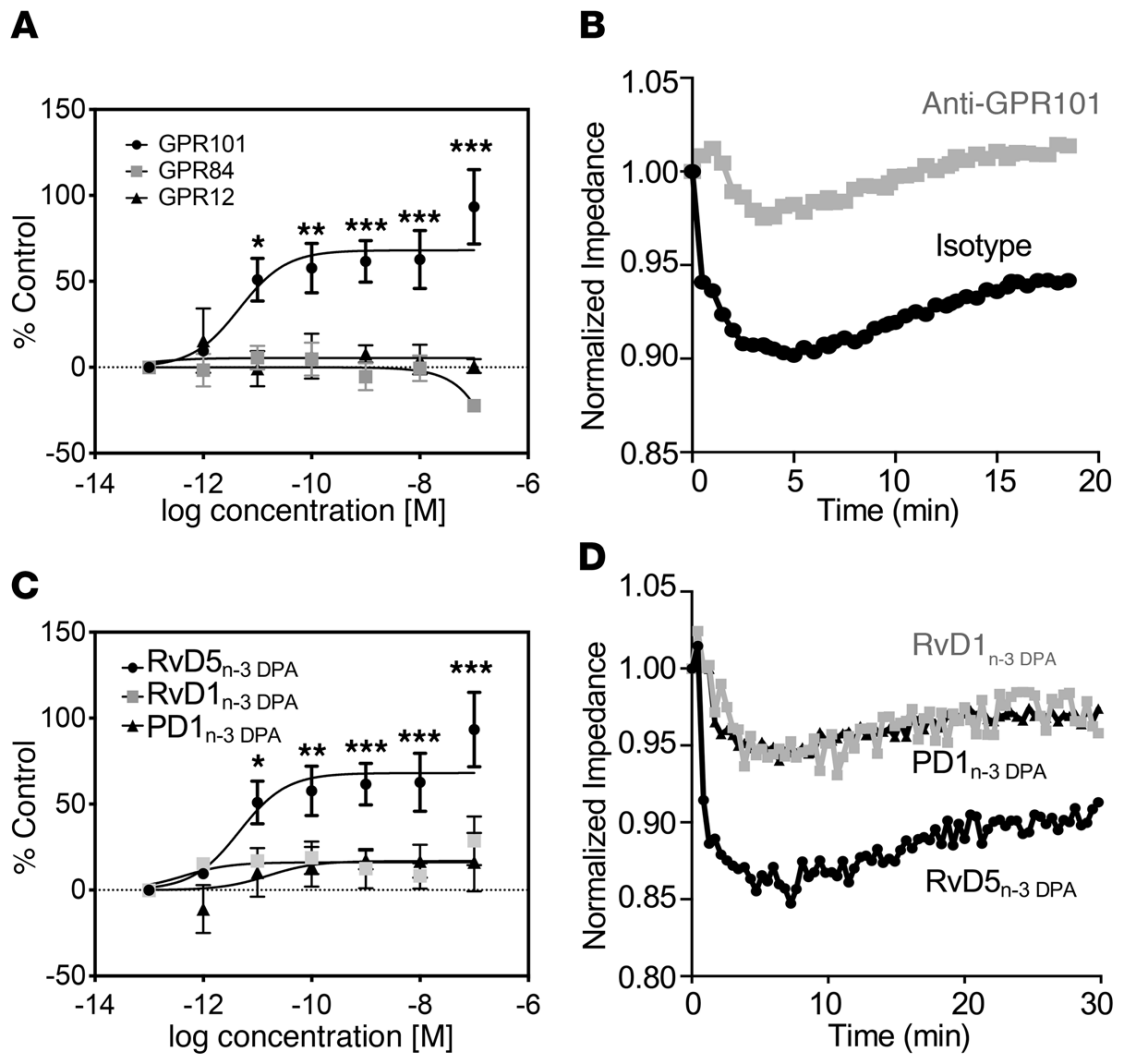
Activation of GPR101 by RvD5_n-3__DPA_. (**A**) RvD5_n-3__DPA_ was incubated at the indicated concentrations with CHO cells expressing human GPR101 (circles), GPR84 (squares), or GPR12 (triangles) coupled with the β-arrestin reporter system, and receptor activation was measured as an increase in luminescence signal. Results represent the mean ± SEM. *n* = 5–7 independent experiments. *P < 0.05, **P < 0.01, and ***P < 0.001 versus the respective vehicle control group; 2-way ANOVA with Tukey’s post hoc multiple comparisons test. (**B**) CHO cells overexpressing GPR101 were incubated with either isotype control or anti-GPR101 antibody (30 minutes at room temperature) and then with 1 nM RvD5_n-3__DPA_, and impedance was measured over a 20-minute period using the xCELLigence DP system. Results are representative of 3 distinct experiments. (**C**) CHO cells expressing GPR101 coupled with the β-arrestin reporter system were incubated with the indicated concentrations of RvD5_n-3__DPA_, RvD1_n-3__DPA_, PD1_n-3__DPA_, or vehicle (PBS containing 0.01% ethanol), and receptor activation was measured as an increase in luminescence signal. Note that the same data is shown for the RvD5_n-3__DPA_ trace as the GPR101 trace in **A**. Results represent the mean ± SEM. *n* = 5–7 independent experiments. *P < 0.05, **P < 0.01, and ***P < 0.001 versus the vehicle control group; 2-way ANOVA with Tukey’s post hoc multiple comparisons test. (**D**) RvD5_n-3__DPA_, RvD1_n-3__DPA_, and PD1_n-3__DPA_ (10 nM) were incubated with GPR101-expressing CHO cells, and impedance was measured over a 30-minute period using the xCELLigence DP system. Results are representative of 3 distinct experiments.

To establish the role of these receptors in mediating the biological actions of RvD5_n-3__DPA_, we evaluated the ability of this ligand to activate each of these 3 receptors using a β-arrestin–based ligand receptor interaction screening system, which enabled the construction of full dose-response curves (19). In these settings, RvD5_n-3__DPA_ increased chemiluminescence in a concentration-dependent manner in cells overexpressing GPR101, with a calculated EC_50_ of 4.6 × 10^–12^ M (Figure 2A). Of note, this increase in chemiluminescence was not observed in cells expressing either GPR12 or GPR84 (Figure 2A). Using the β-arrestin system, we also tested whether RvD5_n-3__DPA_ activates the pro-resolving receptors GPR32 (also known as DRV1) and GPR18 (also known as DRV2). Here, we found that RvD5_n-3__DPA_ displayed an affinity for GPR32/DRV1 comparable to that observed with RvD1, with an EC_50_ of approximately 1.4 × 10^−11^ M and approximately 1.5 × 10^−12^ M, respectively. Of note, RvD5_n-3__DPA_ did not appear to activate GPR18/DRV2 at biologically relevant concentrations (Supplemental Figure 1; supplemental material available online with this article; https://doi.org/10.1172/JCI131609DS1).

Given that GPCR activation leads to changes in both cell shape and size (20), we next used an impedance-based assay to further evaluate the interactions between RvD5_n-3__DPA_ and GPR101. We found that incubation of RvD5_n-3__DPA_ with GPR101-expressing CHO cells led to changes in impedance, an observation that was markedly reversed when these cells were preincubated with an anti-GPR101 antibody (Figure 2B).

**Figure 3:**
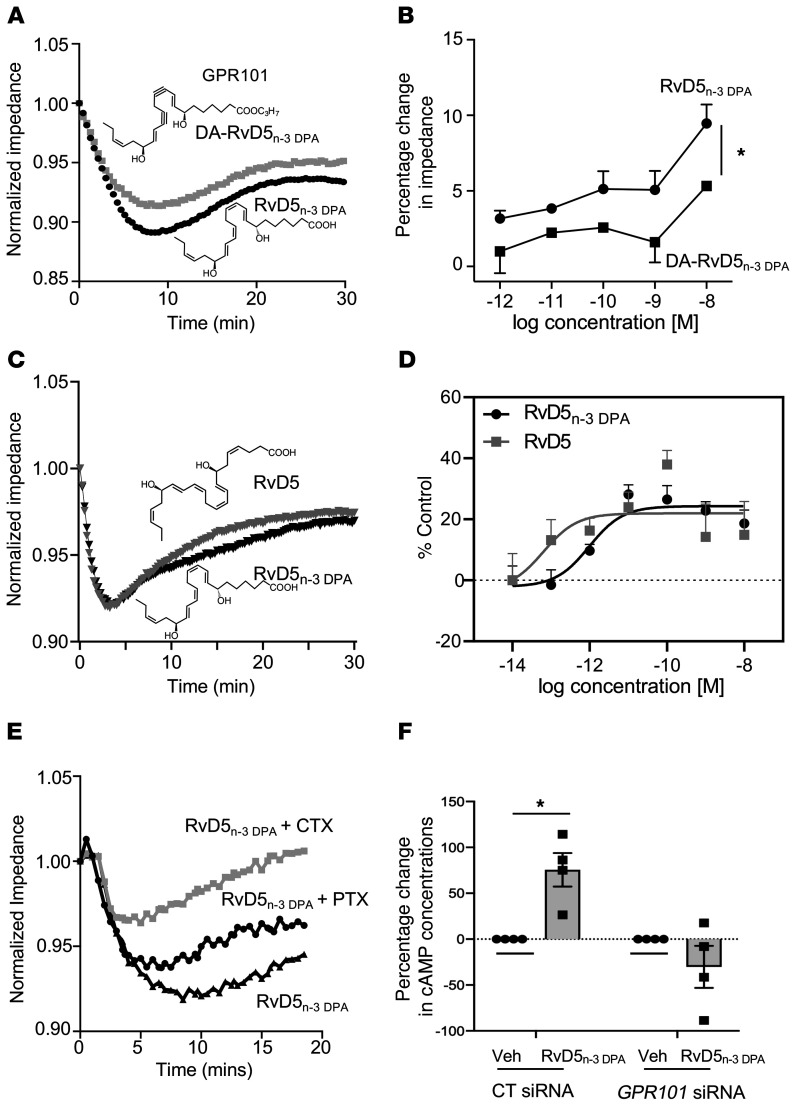
RvD5_n-3__DPA_ stereospecifically activates GPR101 and increases cAMP in human macrophages. (**A**) GPR101-expressing CHO cells were incubated with RvD5_n-3__DPA_ or DA-RvD5_n-3__DPA_ (10 nM), and impedance was measured for 30 minutes. Results are representative of 3 distinct experiments. (**B**) GPR101-expressing CHO cells were incubated with the indicated concentrations of the ligands described in **A**, and changes in impedance from baseline values were determined at *t* = 10 minutes. Results represent the mean ± SEM. *n* = 3 from 3 distinct experiments. *P < 0.05; 1-way ANOVA with a Holm-Sidak post hoc multiple comparisons test. (**C**) GPR101-overexpressing CHO cells were incubated with RvD5_n-3__DPA_ or DHA-derived RvD5 (1 nM), and cell impedance was measured over a 30-minute period using the xCELLigence DP system. Results are shown as the mean ± SEM (*n* = 4 in 3 independent experiments). (**D**) CHO cells expressing GPR101 coupled with the β-arrestin luminescence reporter system were incubated with the indicated concentrations of RvD5_n-3__DPA_, DHA-derived RvD5, or vehicle (cell-plating reagent containing 0.01% ethanol), and receptor activation was measured as an increase in luminescence signal. Results are shown as the mean ± SEM (*n* = 3 in 2 independent experiments). (**E**) GPR101-expressing CHO cells were incubated with CTX (1 μg/mL, 2 hours), PTX (1 μg/mL, 16 hours), or vehicle and then with RvD5_n-3__DPA_ (10 nM), and impedance was measured over a 30-minute period. Results are representative of 3 distinct experiments. (**F**) Human monocyte–derived macrophages were incubated with either an siRNA against *GPR101* or a control sequence (CT siRNA; 72 hours at 37°C) and then with RvD5_n-3__DPA_ (10 nM) or vehicle (Veh) (PBS containing 0.01% ethanol) for 2 minutes, and cAMP concentrations were assessed. Results represent the mean ± SEM (*n* = 4 donors). *P < 0.05; 1-way ANOVA with Holm-Sidak post hoc multiple comparisons test.

We next investigated the stereospecificity of the interaction between RvD5_n-3__DPA_ and GPR101, assessing the capacity of related n-3 DPA–derived SPMs to activate this receptor. Incubation of GPR101-expressing cells with either RvD1_n-3__DPA_ or PD1_n-3 DPA_ (10R,17S-dihydroxy-7Z,11E,13E,15Z,19Z-docosapentaenoic acid) did not elicit notable increases in chemiluminescent signals (Figure 2C), indicating a degree of selectivity for RvD5_n-3__DPA_. We made similar observations when assessing impedance changes, in which addition of RvD1_n-3__DPA_ or PD1_n-3__DPA_ to GPR101-expressing cells only elicited marginal changes in impedance (Figure 2D). To investigate the stereospecific nature of these receptor-ligand interactions, we next tested the ability of isopropyl-(7S,8E,15E,17S,19Z)-7,17-dihydroxydocosa-8,15,19-trien-10,13-diynoate (DA-RvD5_n-3__DPA_), an analog of RvD5_n-3__DPA_, to elicit changes in impedance. We found that substituting the double bond in the middle portion of the analog for triple bond, which would increase the rigidity of the molecule, led to a significant decrease in the ability of the molecule to activate the receptor (Figure 3, A and B). We further evaluated the stereospecific nature of the interaction between RvD5_n-3__DPA_ and GPR101 by assessing whether the DHA-derived RvD5 (7S,17S-dihydroxy-4Z,8E,10Z,13Z,15E,19Z-docosahexaenoic acid), which differs from the n-3 DPA congener by having an extra double bond on carbons 4 and 5, activates GPR101 to a similar degree. Using both impedance assays and the β-arrestin system, we found that RvD5 activated GPR101 to an extent similar to that seen with RvD5_n-3__DPA_, with a calculated EC_50_ of approximately 6.4 × 10^−14^ M and approximately 9.7 × 10^−13^ M, respectively (Figure 3, C and D).

We next tested the role of G proteins in mediating the signals generated by RvD5_n-3__DPA_ via the activation of GPR101. For this purpose, we incubated cells with either cholera toxin (CTX) (G_αs_-like protein inhibitor) or pertussis toxin (PTX) (G_αi_-like protein inhibitor) prior to addition of RvD5_n-3__DPA_. Incubation of cells with CTX, but not PTX, markedly inhibited the changes in impedance elicited by RvD5_n-3__DPA_ (Figure 3E). These results suggest that activation of GPR101 by RvD5_n-3__DPA_ promotes the recruitment of G_αs_-like proteins. Given that cAMP is the downstream second messenger of G_αs_, we next investigated whether RvD5_n-3__DPA_ upregulates cAMP in human monocyte–derived macrophages using an siRNA approach. Incubation of these cells with RvD5_n-3__DPA_ led to an increase in cAMP concentrations (168 ± 56 pmol vs. 290 ± 98 pmol per 0.4 × 10^6^ cells), an increase that was absent in cells transfected with an siRNA against *GPR101* (Figure 3F).

### Binding of ^3^H-RvD5_n-3__DPA_ to GPR101.

**Figure 4:**
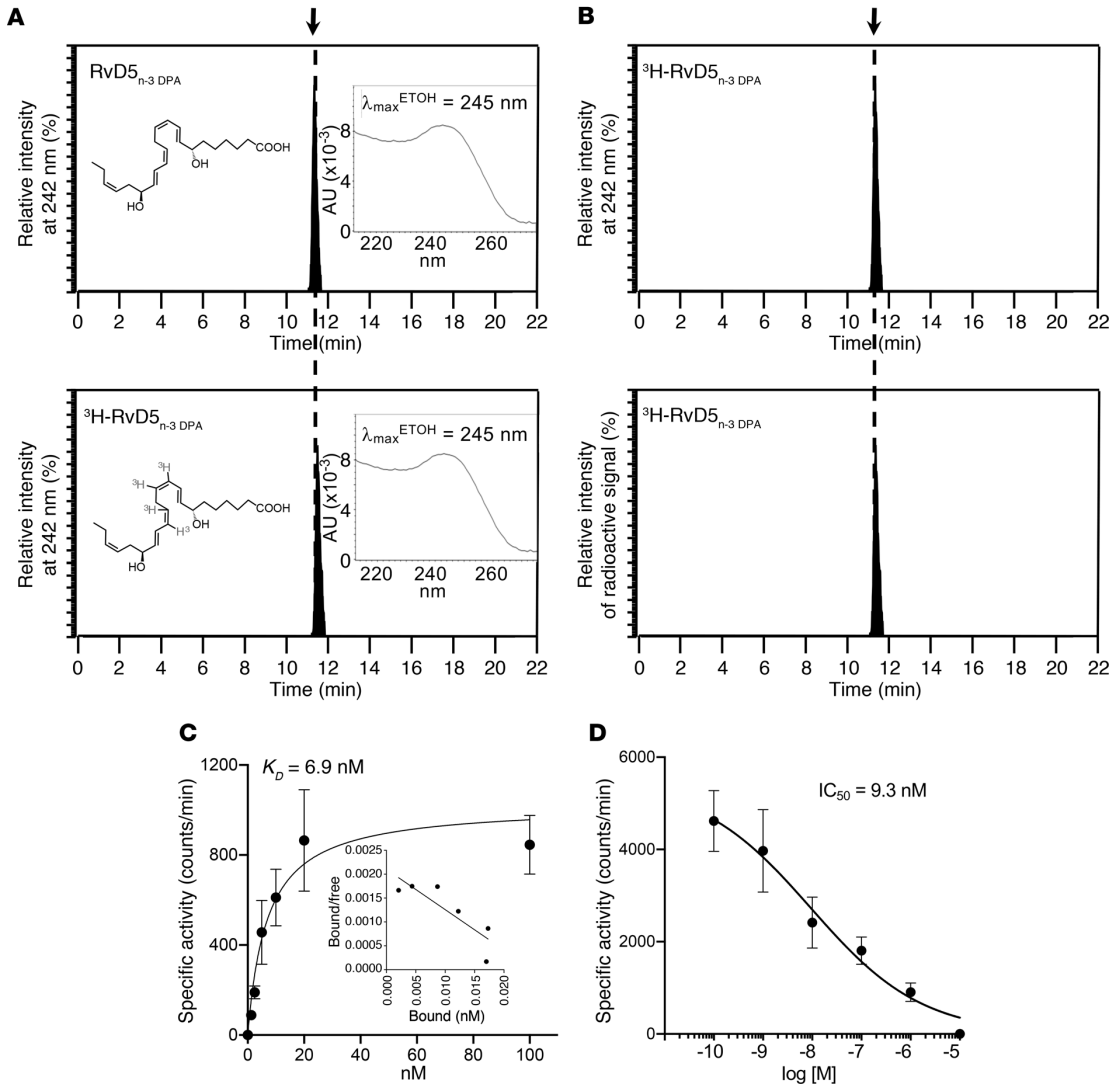
Specific binding of [^3^H]-RvD5_n-3__DPA_ to human GPR101. (**A** and **B**) Characterization of [10, 11, 13, 14 ^3^H]-RvD5_n-3__DPA_ ([^3^H]-RvD5_n-3__DPA_). (**A**) RP-UV-HPLC chromatogram for RvD5_n-3__DPA_ and [^3^H]-RvD5_n-3__DPA_. (**B**) RP-UV-HPLC chromatogram of [^3^H]-RvD5_n-3__DPA_ and online radioactivity monitoring. (**C**) GPR101-overexpressing CHO cells (0.5 × 10^6^ cells in 100 μL) were incubated with [^3^H]-RvD5_n-3__DPA_ at the indicated concentrations in the presence or absence of 10 μM RvD5_n-3__DPA_ (60 minutes at 4°C). Cell incubations were transferred to a vacuum manifold, unbound radioligand was removed, and activity was measured. Results represent the mean ± SEM (*n* = 4 from 2 distinct experiments). Inset shows a Scatchard plot. (**D**) To assess competition binding, GPR101-expressing CHO cells (0.5 × 10^6^ cells in 100 μL) were incubated with 3 nM [^3^H]-RvD5_n-3__DPA_ in the presence or absence of increasing concentrations of RvD5_n-3__DPA_ for 60 minutes at 4°C.

Next, we determined the affinity of the ligand-receptor interaction. For this purpose, we prepared tritium-labeled RvD5_n-3__DPA_ by catalytic tritiation with a specific activity of approximately 40 Ci/mmol. The purity and integrity of the tritiated product were determined using reversed-phase ultraviolet HPLC (RP-UV-HPLC) to be approximately 95%, as demonstrated by essentially identical retention times between the authentic RvD5_n-3__DPA_ and [10, 11, 13, 14^3^H]-RvD5_n-3__DPA_ as well as matching UV chromophores with an UV absorbance maximum in ethanol (λ_max_^ETOH^) of 245 nm (Figure 4A). Furthermore, quantification of radioactivity demonstrated that approximately 98% of the activity was associated with [10, 11, 13, 14^3^H]-RvD5_n-3__DPA_, given that the peak in activity coincided with the eluting of the molecule (Figure 4B). We next conducted saturation binding studies to establish the *K_D_* for binding of RvD5_n-3__DPA_ to GPR101. These experiments yielded a calculated *K_D_* of approximately 6.9 nM (Figure 4C). To determine the specificity of the RvD5_n-3__DPA_-GPR101 interaction, we next sought to establish the IC_50_ of the interaction. We found that RvD5_n-3__DPA_ and [10, 11, 13, 14^3^H]-RvD5_n-3__DPA_ competed for GPR101 binding, with an IC_50_ of approximately 9.3 nM (Figure 4D). Together, these results demonstrate that the affinity of RvD5_n-3__DPA_ for GPR101 was within the bioactive range for this mediator (9, 10).

### GPR101 mediates the regulatory actions of RvD5_n-3__DPA_ on macrophages.

**Figure 5:**
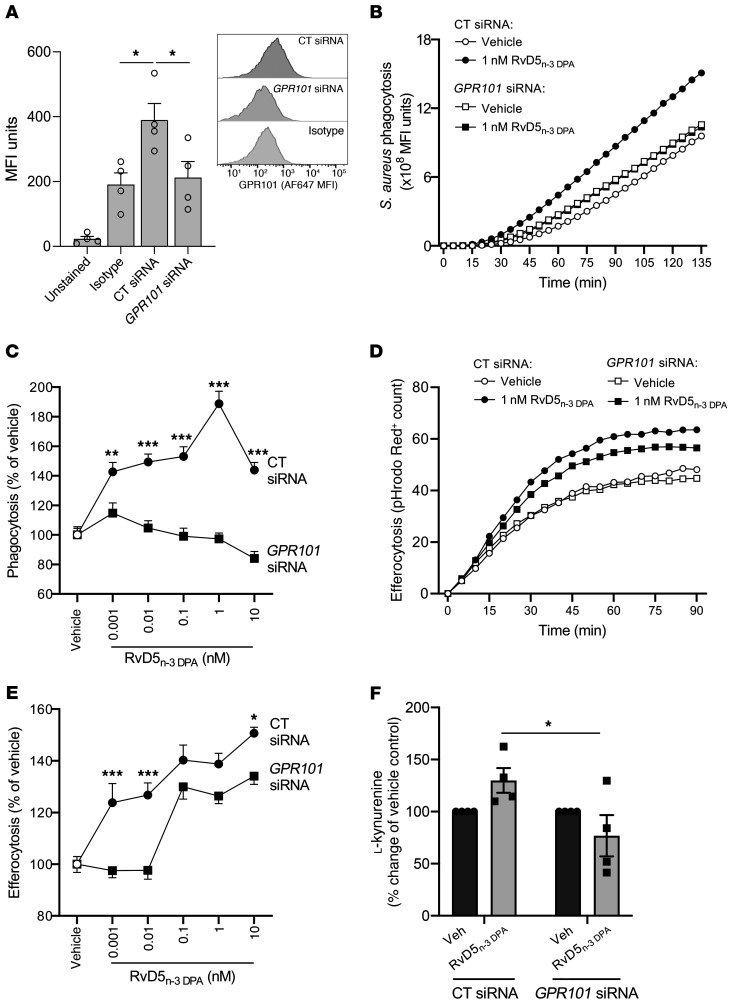
GPR101 mediates the protective actions of RvD5_n-3__DPA_ on human macrophages. (**A**) Human monocyte–derived macrophages were incubated with either an siRNA against *GPR101* or a control sequence (CT siRNA) for 96 hours, and GPR101 expression was assessed using flow cytometry (*n* = 4 donors). (**B**) Cells were transfected as in **A** and then incubated with RvD5_n-3__DPA_ (0.001–10 nM) or vehicle (RPMI-1640 containing 0.1% ethanol, 15 minutes, 37°C), after which (**B** and **C**) efferocytosis of pHrodo Red–conjugated apoptotic HL-60 cells and (**D** and **E**) phagocytosis of pHrodo Green–conjugated *S. aureus* bioparticles were measured using a Zeiss Celldiscoverer 7 high-content imager. **B** and **D** show the increase in signal over time for vehicle and 1 nM RvD5_n-3__DPA_ groups, whereas **C** and **E** show the AUC for all tested concentrations. Results represent the mean ± SEM (*n* = 6 donors from 2 distinct experiments). *P < 0.05, **P < 0.01, and ***P < 0.001; 2-way ANOVA with Tukey’s post hoc multiple comparisons test. (**F**) Human monocyte–derived macrophages were incubated with either an siRNA against *GPR101* or a control sequence and then with 10 nM RvD5_n-3__DPA_ or vehicle (RPMI-1640 containing 0.1% ethanol; 2 hours at 37°C), and the expression of l-kynurenine was measured using LC-MS/MS. Results represent the mean ± SEM (*n* = 4 donors from 2 distinct experiments). *P < 0.05; Friedman’s test with Dunn’s post hoc multiple comparisons test.

In order to test the functional role of GPR101 in mediating the pro-resolving actions of RvD5_n-3__DPA_ in human monocyte–derived macrophages, we next knocked down the expression of this receptor using siRNA. Given the central role of macrophages in promoting the resolution of bacterial infections via the uptake and killing of pathogens in a process termed phagocytosis (4, 7, 18), we next investigated whether RvD5_n-3__DPA_ regulates phagocytosis of bacterial particles in a GPR101-dependent manner. For this purpose, we used pHrodo Green–conjugated *Staphylococcus aureus* (*S. aureus*) bioparticles that become increasingly fluorescent as pH decreases, thus providing a readout of phagocytic uptake and subsequent phagolysosomal degradation of ingested material. Incubation of scrambled control siRNA–transfected macrophages with RvD5_n-3__DPA_ led to a dose-dependent increase in phagocytosis. Transfection of macrophages with an siRNA against *GPR101*, which significantly reduced receptor expression, abrogated the ability of RvD5_n-3__DPA_ to upregulate phagocytosis (Figure 5, A–C).

Next, we tested the ability of these macrophages to take up apoptotic cells, a key pro-resolving action termed efferocytosis (4, 6, 12, 16, 21). Treatment of control siRNA–transfected macrophages with RvD5_n-3__DPA_ led to a dose-dependent increase in efferocytosis of pHrodo Red–labeled apoptotic cells. Meanwhile, transfection of macrophages with an siRNA against *GPR101* limited the ability of RvD5_n-3__DPA_ to upregulate efferocytosis in a similar manner (Figure 5, D and E).

We recently found that RvD5_n-3__DPA_ upregulates the activity of indoleamine 2,3-dioxygenase (IDO) in macrophages (11), which metabolizes tryptophan to l-kynurenine, a mechanism implicated in upregulation of the homeostatic IL-10 receptor (IL-10R) (22). Therefore, we tested the role of GPR101 in mediating the upregulation of l-kynurenine concentrations by monocyte-derived macrophages. In line with published findings, we observed that incubation of control siRNA–transfected macrophages with RvD5_n-3__DPA_ led to an upregulation of l-kynurenine concentrations, whereas addition of this mediator to cells transfected with an siRNA against *GPR101* only marginally regulated the concentrations of this metabolite (Figure 5F). Together, these findings demonstrate a nonredundant role for GPR101 in mediating the biological actions of RvD5_n-3__DPA_ on human monocyte–derived macrophages.

### GPR101 mediates the protective actions of RvD5_n-3__DPA_ on neutrophils and monocytes.

**Figure 6:**
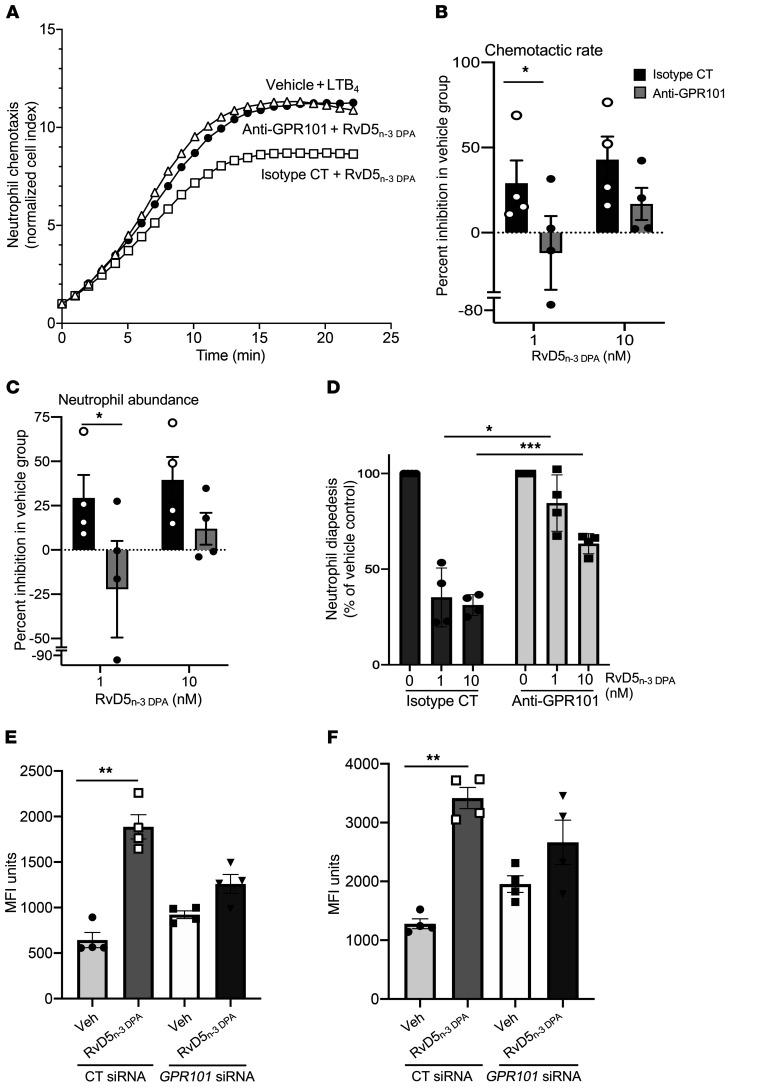
GPR101 mediates the protective actions of RvD5_n-3__DPA_ on neutrophils and monocytes. (**A**–**C**) Human neutrophils were incubated with anti-GPR101 antibody or an isotype control antibody (15 minutes at room temperature) and then with RvD5_n-3__DPA_ at the indicated concentrations or with vehicle (PBS containing 0.1% ethanol), and chemotaxis toward LTB_4_ (10 nM) was assessed using the xCELLigence DP system. (**A**) representative traces, (**B**) neutrophil chemotactic rate calculated from the slope of the curve, and (**C**) neutrophil chemotaxis calculated from the AUC of the traces shown in **A**. *P < 0.05 versus the indicated control group; Kruskal-Wallis test with Dunn’s post hoc multiple comparisons test. (**D**) Neutrophils were isolated and incubated as detailed above, and neutrophil–endothelial cell interactions were assessed after perfusing (0.1 Pa) neutrophils over an activated endothelial cell monolayer. Results represent the mean ± SEM (*n* = 4 donors from 3–4 distinct experiments). *P < 0.05 and ***P < 0.001 versus the indicated control group; 2-way ANOVA with Sidak’s post hoc test. (**E** and **F**) Mice were administered 9 μg siRNA against mouse *Gpr101* or a scrambled control sequence. After 72 hours, blood was collected and cells incubated with RvD5_n-3__DPA_ (10 nM) or vehicle (20 minutes at 37°C) and then with 5 × 10^7^ CFU fluorescently labeled bacteria (30 minutes at 37°C), and phagocytosis in (**E**) neutrophils and (**F**) monocytes was assessed by flow cytometry. Results represent the mean ± SEM (*n* = 4 per group from 2 distinct experiments). **P < 0.01 versus the indicated control group; Kruskal-Wallis test with Dunn’s post hoc multiple comparisons test.

Since RvD5_n-3__DPA_ regulates the biological actions of neutrophils (9, 10, 23) and GPR101 is expressed on these cells (Figure 1), we complemented the results produced with human monocyte–derived macrophages by determining the neutrophil-directed actions of RvD5_n-3__DPA_. Incubation of RvD5_n-3__DPA_ with human neutrophils led to a significant and dose-dependent reduction in both the rate of neutrophil migration toward leukotriene B_4_ (LTB_4_) and the total number of cells migrated (Figure 6, A–C). In addition, RvD5_n-3__DPA_ also reduced the number of cells that transmigrated through an activated endothelial cell monolayer under flow conditions (Figure 6D). Incubation of neutrophils to an antibody targeting GPR101 reversed the biological actions of RvD5_n-3__DPA_, increasing both neutrophil chemotaxis and transmigration (Figure 6, A–D).

Since neutrophils are pivotal for the clearance of bacterial infections (3, 4), we next tested whether RvD5_n-3__DPA_, via GPR101, increases the ability of these cells to take up bacteria. Flow cytometric assessment of peripheral blood neutrophils and monocytes demonstrated that these cells expressed the murine ortholog of human GPR101 (Supplemental Figure 2). Incubation of whole blood from mice transfected with a control siRNA sequence with RvD5_n-3__DPA_ led to a significant increase in bacterial phagocytosis by both neutrophils (643 ± 96 vs. 1886 ± 154 MFI units) and monocytes (1280 ± 97 vs. 3418 ± 208 MFI units). These actions were reversed when the mediator was added to peripheral blood collected from mice transfected with siRNA against *Gpr101* that reduced the expression of the receptor on peripheral blood leukocytes (Figure 6, E and F, and Supplemental Figure 3). Together, these findings establish a role for GPR101 in mediating the biological actions of RvD5_n-3__DPA_ on neutrophils and monocytes.

### GPR101 mediates the antiarthritic actions of RvD5_n-3 DPA_.

**Figure 7:**
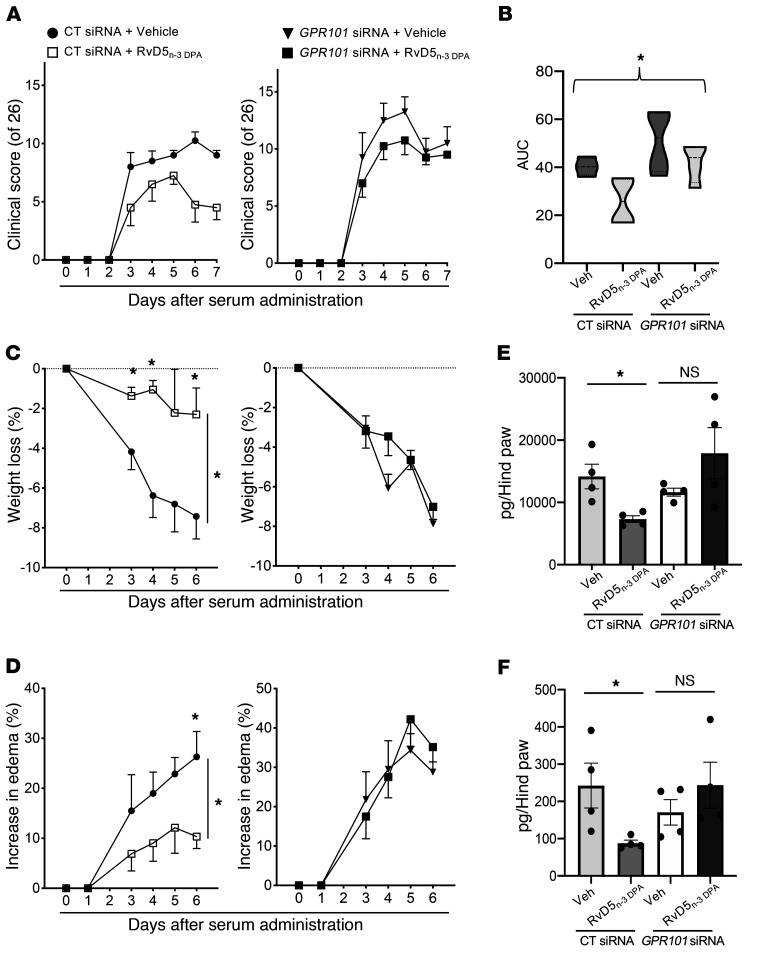
Knockdown of GPR101 reverses the antiarthritic actions of RvD5_n-3__DPA_. Mice were administered 9 μg siRNA targeting mouse *Gpr101* or a scrambled control sequence. After 24 hours and 72 hours, mice were administered arthritogenic serum and then treated with RvD5_n-3__DPA_ (150 ng/mouse) or vehicle (72 hours and 96 hours after siRNA administration). (**A**) Clinical scores, (**B**) AUC for clinical scores, (**C**) weight loss, and (**D**) edema were determined throughout the disease process. (**E** and **F**) On day 7, hind paws were harvested and eicosanoid concentrations determined using LC-MS/MS–based lipid mediator profiling. (**E**) Prostaglandin and (**F**) LTB_4_ metabolomic concentrations. Results represent the mean ± SEM (*n* = 4 mice per group). *P < 0.05 versus the vehicle-treated group; Kruskal-Wallis test with Dunn’s post hoc multiple comparisons test (**B**, **E**, and **F**) and 2-way ANOVA (**C** and **D**).

RvD5_n-3__DPA_ displays potent antiarthritic actions that regulate both neutrophil and macrophage responses in inflammatory arthritis (11). Thus, we next investigated the role of GPR101 in mediating these protective actions. For this purpose, we treated mice with either an siRNA targeting mouse *Gpr101* to knock down the expression of this receptor in vivo, or a control sequence. We then challenged the mice with arthritogenic serum from K/BxN mice, which initiates a polyarthritic disease driven by both monocytes and neutrophils (24). Assessment of clinical scores revealed that treatment of mice transfected with a control siRNA sequence with RvD5_n-3__DPA_ led to a significant reduction in clinical scores when compared with vehicle-treated mice (5.5 ± 1.8 in RvD5_n-3__DPA_–treated mice vs. 9.0 ± 0.5 in mice given vehicle), whereas in mice transfected with an siRNA against *Gpr101*, this antiarthritic activity of RvD5_n-3 DPA_ was lost (9.5 ± 0.3 in RvD5_n-3 DPA_ –treated mice vs. 10.5 ± 1.7 in mice given vehicle) (Figure 7, A and B). Transfection of mice with an siRNA against *Gpr101* diminished the ability of this mediator to protect against weight loss (a marker of disease severity), limit joint edema, and reduce joint concentrations of the inflammation-initiating prostaglandins and LTB_4_ (Figure 7, C–F).

We recently found that inflammatory arthritis disrupts gut barrier function, which promotes joint inflammation (11). RvD5_n-3 DPA_ administration reduced intestinal inflammation and eicosanoid concentrations and upregulated intestinal pro-resolving mediator levels (11). Therefore, in this study, we assessed whether GPR101 was central to these modulatory properties of RvD5_n-3__DPA_. Lipid mediator profiling of intestinal tissues demonstrated that in mice administered a control siRNA sequence, RvD5_n-3__DPA_ reduced the concentrations of the inflammatory eicosanoids, including PGD_2_ (193.4 ± 38.9 in vehicle-treated mice vs. 95.0 ± 15.7 pg/50 mg tissue) and PGE_2_ (376.3 ± 58.3 in vehicle-treated mice vs. 177.8 ± 16.9 pg/50 mg tissue), and increased the concentrations of select SPMs, namely RvD4 (0.6 ± 0.24 in vehicle-treated-mice vs. 2.7 ± 0.3 pg/50 mg tissue) and 17R-RvD1 (0.0 ± 0.0 in vehicle-treated mice vs. 0.21 ± 0.05 pg/50 mg tissue). Of note, these protective actions were diminished in mice transfected with the siRNA against *Gpr101* prior to RvD5_n-3__DPA_ administration (Supplemental Figure 4).

### RvD5_n-3 DPA_ accelerates the resolution of bacterial infections via GPR101.

**Figure 8:**
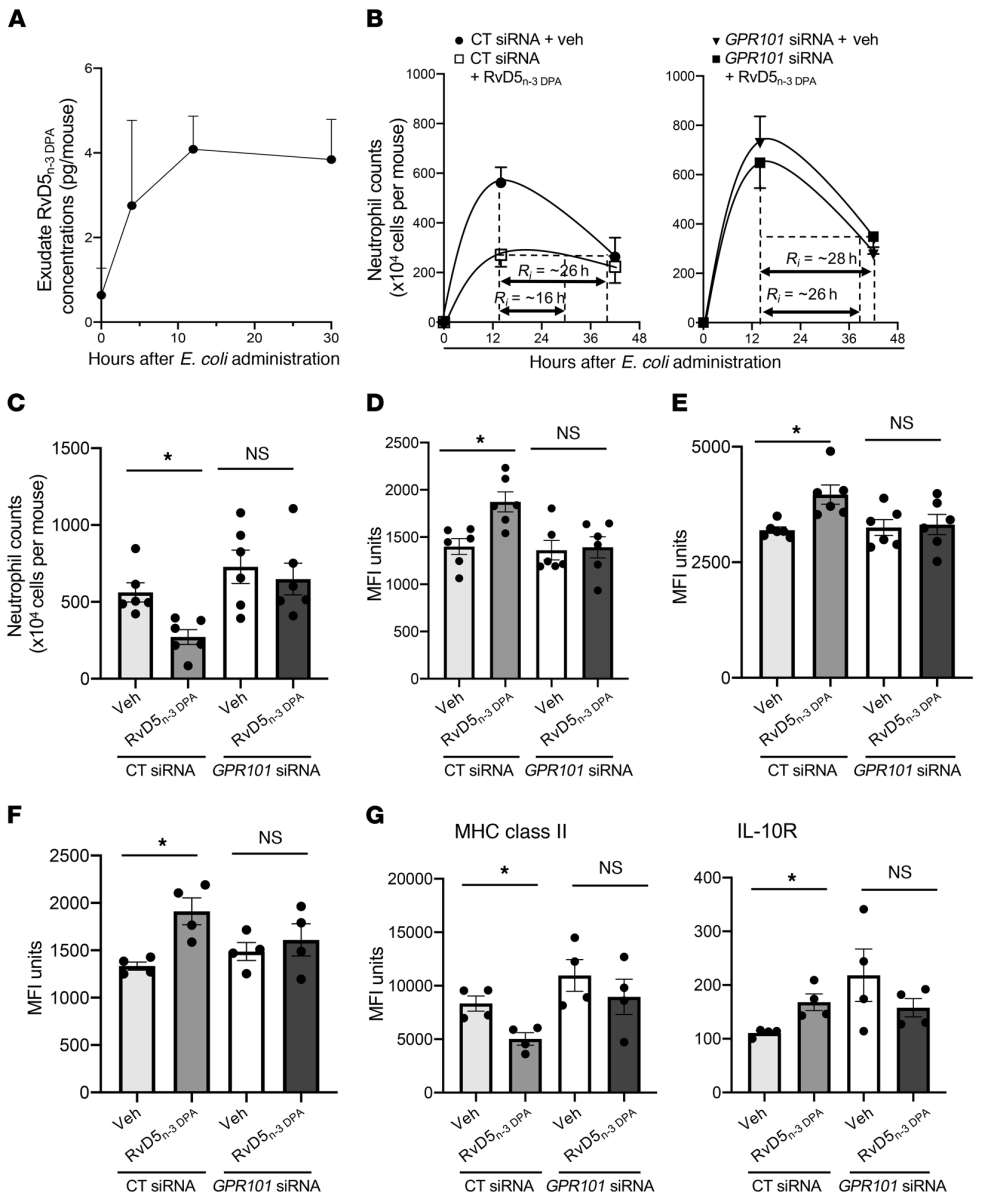
*Gpr101* knockdown limits the ability of RvD5_n-3__DPA_ to activate host responses to promote the resolution of *E.* coli infections. (**A**) Mice were inoculated with 10^5^ CFU *E*. *coli* via i.p. injection and lavages collected at the indicated time intervals before (0 hours) or after inoculation. RvD5_n-3__DPA_ concentrations were determined using lipid mediator profiling. Results represent the mean ± SEM (*n* = 3 mice per group). (**B**) Mice were administered 9 μg siRNA against mouse *Gpr101* or a scrambled control sequence, and after 72 hours, they were administered RvD5_n-3__DPA_ (100 ng/mouse) or vehicle control (PBS containing 0.1% ethanol) and then inoculated with 10^5^ CFU *E*. *coli* via i.p. injection. (**C**) Fourteen-hour exudate neutrophil counts. Results represent the mean ± SEM (*n* = 6 mice per group from 2 distinct experiments). (**D** and **E**) Bacterial phagocytosis was determined in exudate (**D**) neutrophils and (**E**) macrophages at the 14-hour interval using flow cytometry. (**F**) Efferocytosis was determined at the 14-hour interval in CD64^+^F4/80^+^ macrophages using flow cytometry. (**G**) The expression of MHC class II and IL-10R was assessed in CD64^+^F4/80^+^ macrophages using flow cytometry. Results represent the mean ± SEM (*n* = 6 mice per group from 2 distinct experiments). *P < 0.05 versus the vehicle-treated group; Kruskal-Wallis test with Dunn’s post hoc multiple comparisons test (**C**–**G**).

Having established that RvD5_n-3 DPA_ increased the ability of monocytes, macrophages, and neutrophils to take up bacteria, we next questioned whether this mediator was produced during bacterial infections and whether its production was temporally regulated during self-limited infections. For this purpose, we inoculated mice with *E*. *coli* and assessed the temporal production of RvD5_n-3 DPA_ using lipid mediator profiling. This analysis demonstrated that RvD5_n-3 DPA_ was produced during bacterial infections, with its production reaching a maximum at the 12-hour interval and remaining sustained into the resolution phase of the inflammatory response (Figure 8A). We next tested whether the protective actions of RvD5_n-3__DPA_ in promoting bacterial clearance as observed in vitro were retained in vivo and examined the role of GPR101 in mediating the observed regulatory actions. In mice given a control siRNA sequence, administration of RvD5_n-3__DPA_ at a dose as low as 100 ng/mouse accelerated the resolution of *E*. *coli*–initiated inflammation, reducing the resolution interval (R_i_) (i.e., the time it takes for neutrophil numbers to reduce from peak values to half maximum) from 26 hours to 16 hours (Figure 8B). This reduction in the resolution interval was linked with a significant reduction in the number of neutrophils recruited to the peritoneum (5.6 ± 0.9 × 10^6^ in vehicle-treated mice vs. 2.7 ± 0.7 × 10^6^ cells per mouse in RvD5_n-3__DPA_–treated mice) (Figure 8C); a significant upregulation in the ability of exudate neutrophils (1400 ± 118 MFI units in vehicle-treated mice vs. 1873 ± 150 MFI units in RvD5_n-3__DPA_–treated mice) and macrophages (3195 ± 94.7 MFI units in vehicle-treated mice vs. 3965 ± 292 MFI units in RvD5_n-3__DPA_–treated mice) to take up bacteria (Figure 8, D and E); and macrophage efferocytosis (Figure 8F). In these exudates, we also found a significant reduction in PGD_2_, PGE_2_ and the LTB_4_ metabolome, which encompasses the neutrophil chemoattractant LTB_4_ (Supplemental Figure 5).

Since macrophages are central players in both the termination and propagation of acute inflammation (4, 7, 18), we next investigated whether RvD5_n-3__DPA_ regulates exudate macrophage phenotype during infectious inflammation. In exudate macrophages from mice given RvD5_n-3__DPA_ and a control siRNA, we observed a decrease in the expression of MHC class II molecules (4863 ± 542 MFI units vs. 9136 ± 1536 MFI units in RvD5_n-3__DPA_–treated mice vs. vehicle-treated mice). We observed an increase in the expression of the fractalkine receptor CX3CR1 (4010 ± 198 MFI units vs. 3160 ± 148 MFI units in RvD5_n-3__DPA_–treated mice vs. vehicle-treated mice); the lipid mediator biosynthetic enzyme COX-2 (994 ± 70 MFI units vs. 1317 ± 106 MFI units in RvD5_n-3__DPA_–treated mice vs. vehicle-treated mice); and the IL-10R (195 ± 35 MFI units vs. 88 ± 20 MFI units in RvD5_n-3__DPA_–treated mice vs. vehicle-treated mice) (Figure 8G and Supplemental Figure 6), markers associated with a homeostatic and resolution macrophage phenotype (25, 26). Of note, transfection of mice with an siRNA against mouse *Gpr101*, which led to a reduction in neutrophil and macrophage receptor expression (Supplemental Figure 7), abolished the protective actions of RvD5_n-3__DPA_ on both of these cell types (Figure 8, A–G, and Supplemental Figure 7). Together, these findings demonstrate that RvD5_n-3__DPA_ activates host innate immune responses to promote the termination of infectious inflammation, actions that are mediated by GPR101.

## Discussion

In the present study, using a screening approach, we identified GPR101 as a receptor for RvD5_n-3__DPA_. We found that RvD5_n-3__DPA_ bound and activated GPR101 at a concentration that was commensurate with its biological actions. Inhibition of GPR101 using anti-GPR101 antibodies and siRNA demonstrated a role for this receptor in mediating the immunoregulatory actions of RvD5_n-3__DPA_ in both human and mouse leukocytes. Knockdown of *Gpr101* in vivo reversed the joint protective actions of RvD5_n-3 DPA_ during inflammatory arthritis and the pro-resolving actions of this mediator during bacterial infections.

GPR101 is an X-linked receptor expressed on chromosome Xq26.3, the longer arm of the X chromosome. The GPR101 gene encodes a 508-aa deduced protein that shares approximately 30% sequence homology in the transmembrane regions with the α-1A-adrenergic receptor and the serotonin 5HT1A receptor (https://www.omim.org/entry/300393#1). In human brain tissues, two GPR101 transcript variants have been identified, one of 9.5 kb and the second of 4.2 kb, whereas in mouse brain, a 5.5-kb transcript was identified. The human GPR101 protein shares approximately 71% identity with the deduced mouse protein, which is predicted to be 511 aa in size. In the present study, we demonstrate that this receptor was expressed on human and mouse neutrophils, monocytes, and macrophages (Figure 1 and Supplemental Figures 2, 3, and 7). In humans, several mutations have been identified for this receptor that include the E308D mutation, which was identified in tumour DNA (27). A second mutation, the substitution of D366E, was identified in a patient with sporadic acromegaly (28). However, the functional impact of these mutations is not well characterized, given that the endogenous ligand(s) for this receptor were not known.

Pro-resolving mediators exert potent actions in the regulation of innate immune responses. These biological actions are mediated via activation of select GPCRs, to which these molecules bind with high stereospecificity and affinity (14–18). In the present study, using radiolabeled RvD5_n-3__DPA_, we found that this mediator bound to GPR101 with a *K_D_* of approximately 6.9 nM, which is comparable to its bioactive concentration range. We also found that RvD5_n-3__DPA_ at concentrations as low as 0.1–10 nM was able to activate the receptor. This ability to activate intracellular signaling was found to be stereospecific, since neither RvD1_n-3__DPA_ nor PD1_n-3__DPA_ elicited marked changes in luminescence or impedance in GPR101-transfected CHO cells. Furthermore, subtle changes to the chemical backbone such as the substitution of the double bond at C10 and C13 for triple bond, which increases the rigidity of the molecule, also led to a significant (*P* < 0.5) decrease in its ability to activate the receptor.

Flow cytometric analysis revealed that GPR101 was expressed on both human and mouse neutrophils, monocytes, and macrophages. Leukocytes are involved in the initiation and termination of both sterile and inflammatory responses, with neutrophils, monocytes, and macrophages being the first responders (1, 2, 6, 7, 9, 18). SPMs, including RvD5_n-3 DPA_, have leukocyte-directed actions that regulate leukocyte trafficking to the site of inflammation (4, 8, 10, 18), promoting the uptake and killing of bacteria and enhancing the clearance of apoptotic cells and cellular debris. Results from the present studies demonstrate that knockdown of GPR101 limited the ability of human macrophages to take up both apoptotic cells and bacteria in response to RvD5_n-3 DPA_. Furthermore, inhibition of the receptor on human neutrophils using an anti-GPR101 antibody limited the biological actions of RvD5_n-3__DPA_, thereby reducing the ability of this mediator to promote the uptake and killing of bacteria, reversing its antichemotactic actions on neutrophils, and limiting its ability to reduced neutrophil diapedesis.

Rheumatoid arthritis is an autoimmune disease perpetuated by unremitting joint inflammation that results in bone destruction and joint deformation (29). Neutrophils and monocytes are implicated in the propagation of the effector phase of the disease. In a recent study by our group, we found that, in addition to joint inflammation, inflammatory arthritis also led to gut inflammation and weakening of the gut barrier, an observation that was linked with a reduction in intestinal levels of RvD5_n-3__DPA_ (11). Furthermore, administration of RvD5_n-3__DPA_ to arthritic mice reduced both joint and gut inflammation, restoring barrier function and reducing leukocyte infiltration into the paws (9). Here, we report that knockdown of GPR101 limited the protective actions of RvD5_n-3__DPA_ in reducing joint and intestinal inflammation in experimental inflammatory arthritis. Knockdown of this receptor also blunted the ability of RvD5_n-3__DPA_ to reduce the levels of inflammatory eicosanoids in the gut and joints of arthritic mice as well as promote the upregulation of SPMs in arthritic mice.

Bacterial infections result from an inability of the host immune response to contain invading pathogens (1). The prevailing approach to treating infections is to inhibit bacterial replication using antibiotics. The recent rise in antibiotic-resistant bacteria has spurred the search for novel approaches to treat infections. Studies investigating endogenous mechanisms promoting the resolution of bacterial infections demonstrate that SPMs reprogram the host immune response to positively regulate the uptake and killing of bacteria (4). Indeed, we found here that RvD5_n-3__DPA_ promoted the uptake of bacteria by neutrophils, monocytes, and macrophages in a GPR101-dependent manner. Administration of RvD5_n-3__DPA_ to mice also reduced the exudate concentrations of inflammatory eicosanoids, neutrophil infiltration into the site of inflammation, and the resolution interval from 26 hours to 16 hours. Of note, knockdown of GPR101 reversed these protective actions, underscoring the role of SPMs in reprogramming host immune responses during infection and the role of GPR101 in mediating the immunomodulatory actions of RvD5_n-3__DPA_ during infections.

In summary, we have identified an immunoregulatory axis centered on GPR101/RvD5_n-3__DPA_ that promotes the termination of both sterile and infectious inflammation. These findings demonstrate that GPR101 is the conveyor for the immune-regulatory properties of RvD5_n-3__DPA_ and establish a role for this receptor in mediating the regulation of joint inflammation, both locally and remotely through the gut barrier, and in instructing the host to efficiently dispose of bacterial pathogens. Thus, these results provide new cues for harnessing the immune-modulatory actions of pro-resolving mediators by developing innovative therapeutic approaches based on the RvD5_n-3__DPA_/GPR101 axis.

## Methods

### GPCR screening

Using PathHunter β-arrestin enzyme fragment complementation (EFC) technology coupled with β-gal (DiscoveRx), we screened 49 orphan receptors. GPCR screening was performed by incubating 10 nM RvD5_n-3__DPA_ or vehicle control (PBS in 0.01% ethanol) at 37°C for 90 minutes with the candidate GPCRs, and receptor activation was determined by measuring the luminescence signal. Negative controls were used to measure the potential constitutive activity in the absence of ligand. This custom screening was performed in duplicate, and the mean chemiluminescence value was used for analysis. The percentage of activity was calculated using the following formula: percentage of activity = 100% × (mean relative luminescence units [RLU] of the test sample – mean RLU of the vehicle control)/(mean RLU of the vehicle control).

### GPCR–β-arrestin assays

To monitor downstream receptor activation, the β-arrestin PathHunter system (DiscoveRx) was used. CHO cells stably expressing human recombinant GPR12, GPR84, or GPR101 coupled to a β-arrestin reporter system were cultured in media containing 10% heat-inactivated FBS. GPCR and β-arrestin expression was maintained by dual selection in hygromycin B and geneticin according to the manufacturer’s instructions (DiscoveRx). For the experiments, single-use (eXpress) CHO cells stably expressing human recombinant GPR18 or GPR32 coupled to a β-arrestin reporter system were used. For all GPCR–β-arrestin assays, cells were plated in 96-well plates 48 hours before experiments using AssayComplete Cell Plating Reagent (DiscoveRx). Cells were incubated with the indicated concentrations of RvD5_n-3__DPA_ (Vinresol, custom synthesis), PD1_n-3__DPA_, or RvD1_n-3__DPA_ (provided by Trond Vidar Hansen, University of Oslo, Oslo, Norway) for 1 hour at 37°C, and receptor activation was determined by measuring chemiluminescence using the PathHunter Detection Kit (DiscoveRx).

### xCELLigence RTCA DP system

Ligand-receptor interactions were assessed by monitoring impedance changes across single-cell monolayers using an xCELLigence DP System (ACEA Biosciences). GPR101-overexpressing CHO cells were plated on electronic microtiter plates (E-Plates). Test compounds were added to the chambers in serum-free medium, and real-time impedance changes were monitored (0–30 minutes at 37°C). For antibody incubations, anti-GPR101 antibody (clone G278, host: rabbit, Assay Biotechnology) or nonimmune rabbit IgG was incubated with cells on microtiter plates at 1:50 dilutions for 20 minutes before addition of the indicated mediators. In select experiments, GPR101-overexpressing CHO cells were incubated with CTX (1 μg/mL, 2 hours), PTX (1 μg/mL, 16 hours), or vehicle. Cells were then washed with serum-free HAM F-12 serum-free medium. RvD5_n-3__DPA_ (10 nM) was added and impedance changes were assessed over a 30-minute period.

### Preparation of [10, 11, 13, 14^3^H]-RvD5_n-3__DPA_ and radioligand binding

Total organic synthesis of [10, 11, 13,14^3^H]-RvD5_n-3 DPA_ was done by Vinresol (custom synthesis) via the tritiation of (7S,8E,15E,17S,19Z)-7,17-dihydroxydocosa-8,15,19-trien-10,13-diynoate. The integrity and activity of the radioligand was ascertained using reverse-phase UV-HPLC (RV-UV-HPLC) system (1100 Series, Agilent Technologies) equipped with an Eclipse Plus C18 Column (100 mm × 4.6 mm × 1.8 μm; Agilent Technologies) coupled with a diode array detector (DAD) (G1315B; Agilent Technologies). A gradient of methanol/water of 55:45 (vol/vol) was ramped to 63:37 (vol/vol) over a 22-minute period and then to 98:2 (vol/vol) for the next 8 minutes. The flow rate was maintained at 0.5 mL/min.

To assess the affinity of the radioligand for human GPR101, GPR101-overxpressing CHO cells were suspended in DPBS containing Ca^2+^ and Mg^2+^. For competition binding experiments, 0.5 × 10^6^ cells/0.1 mL were incubated with approximately 3 nM [10, 11, 13,14^3^H]-RvD5_n-3__DPA_ (specific activity ~40 Ci/mmol) in the absence or presence of increasing concentrations of unlabeled RvD5_n-3__DPA_ (1 nM–10 μM). Following incubation for 60 minutes at 4°C, cells were transferred to Whatman GF/C glass microfiber filters (Thermo Fisher Scientific), and unbound ligand was removed by washing twice with 4 mL ice-cold Dulbecco’s PBS (DPBS) using a vacuum manifold (MilliporeSigma). Bound radioactivity was then determined using a scintillation counter (Beckman Coulter). Nonspecific binding was determined in the presence of 10 μM unlabeled RvD5_n-3__DPA_.

For saturation binding experiments, 0.5 × 10^6^ cells/0.1 mL cells were incubated with [10, 11, 13,14^3^H]-RvD5_n-3__DPA_ (1.25–100 nM), and then with or without 10 μM RvD5_n-3__DPA_ for 60 minutes at 4°C. Cells were then transferred onto Whatman GF/C glass microfiber filters, and unbound ligand was removed by washing twice with 4 mL ice-cold DPBS using a vacuum manifold (MilliporeSigma). Bound radioactivity was determined using a scintillation counter (Beckman Coulter).

### GPCR expression on primary cells

#### Human leukocyte GPCR expression.

Expression of GPR101, GPR12 (host: rabbit, lot AG11012912, catalog bs-13514R, Bioss) and GPR84 (host: mouse, lot 08248 WECz, catalog H00053831-A01, Abnova) was assessed in human peripheral blood monocytes, neutrophils, and macrophages. Nonspecific binding was quenched using FC blocking solution. Human cells were incubated with rabbit anti–human GPR101, rabbit anti–human GPR12, or mouse anti–human GPR84 (30 minutes at 4°C) and then with AF488-conjugated goat anti–rabbit IgG (Thermo Fisher Scientific) or AF488-conjugated goat anti–mouse IgG (Thermo Fisher Scientific) for 30 minutes at 4°C. Cells were then incubated with AF647-conjugated anti–human CD14 (clone HCD14, BioLegend) and APC-Cy7–conjugated anti–human CD16 (clone 3G8, BioLegend; for monocytes) antibodies (30 minutes at 4°C). For whole blood incubation, RBCs were lysed using a BD Pharmingen Red Blood Cell Lysis Kit following the manufacturer’s instructions, and fluorescence staining was evaluated using an LSR Fortessa Cell Analyzer (BD Biosciences) and FlowJo software, version 10 (Tree Star).

#### Mouse leukocyte GPCR expression.

Peripheral blood was collected via cardiac puncture in 3.2 % sodium citrate. Cells were then incubated with anti–mouse CD16/CD32 (clone 93, eBioscience; dilution 1:100, 20 minutes at 4°C in PBS containing 5% FCS staining solution). Peripheral blood cells were then incubated with AF700-conjugated anti–mouse Ly6G (clone 1A8, BioLegend; for neutrophils) and BV421-conjugated anti–mouse CD115 (clone AFS98, BioLegend; for monocytes) antibodies (30 minutes at 4°C), and receptor expression was evaluated as described above.

### Targeted lipid mediator profiling

Paws and intestinal tissues were collected and immediately transferred to liquid nitrogen prior to homogenization in 1 mL ice-cold MeOH using a glass dounce. All samples for LC-MS/MS–based profiling were extracted using solid-phase extraction columns as described previously (30). Prior to sample extraction, deuterated internal standards, representing each region in the chromatographic analysis (500 pg each) were added to facilitate quantification. Samples were kept at –20°C for a minimum of 45 minutes to allow for protein precipitation. Supernatants were subjected to solid-phase extraction, and the methyl formate fraction was collected, brought to dryness, and suspended in phase (methanol/water, 1:1, vol/vol) for injection on a Shimadzu LC-20AD HPLC and a Shimadzu SIL-20AC autoinjector, paired with a QTrap 6500 Plus (Sciex). An Agilent Poroshell 120 EC-C18 column (100 mm × 4.6 mm × 2.7 μm) was kept at 50°C and the mediators eluted using a mobile phase consisting of methanol/water/acetic acid ratio of 20:80:0.01 (vol/vol/vol) that was ramped to 50:50:0.01 (vol/vol/vol) over 0.5 minutes and then to 80:20:0.01 (vol/vol/vol) from 2 minutes to 11 minutes, maintained until 14.5 minutes, and then rapidly ramped to 98:2:0.01 (vol/vol/vol) for the next 0.1 minutes. This was subsequently maintained at 98:2:0.01 (vol/vol/vol) for 5.4 minutes, and the flow rate was maintained at 0.5 mL/min. QTrap 6500 Plus was operated using a multiple reaction monitoring method as previously described (30). Each lipid mediator was identified using established criteria including matching of the retention time to synthetic or authentic standards and at least 6 diagnostic ions (30). Calibration curves were obtained for each mediator using lipid mediator mixtures at 0.78, 1.56, 3.12, 6.25, 12.5, 25, 50, 100, and 200 pg that gave linear calibration curves with *r^2^* values of 0.98–0.99.

### Animal experiments

#### E. coli peritonitis.

Six- to eight-week-old male C57BL/6 mice (Charles River Laboratories, the supplier fed the mice a lab diet containing essential fatty acids) were administered 9 μg Accell Control siRNA or Accell Mouse GPR101 siRNA SMARTpool (Dharmacon) via i.p. injection. After 3 days, mice were administered RvD5_n-3__DPA_ (100 ng/mouse) or vehicle via i.p. injection immediately prior to i.p. injection of live *E*. *coli* (serotype O6:K2:H1, 10^5^ CFU per mouse). At designated time intervals, the mice were euthanized, blood was collected via cardiac puncture in sodium citrate, and peritoneal exudates were collected in 4 mL PBS.

The cellular composition in the exudates was determined using Turk’s solution, light microscopy, and flow cytometry. For the latter, exudate cells were incubated with anti–mouse CD16/CD32 (clone 93, eBioscience; dilution 1:100, 20 minutes at 4°C in PBS containing 5% FCS staining solution), followed by incubation with phycoerythrin (PE) CD64 (FcγRI, clone X54-5/7.1, host mouse, BioLegend), Alexa Fluor 700 Ly-6G (clone 1A, host rat, BioLegend), Brilliant Violet 711 CD115 (CSF-1R) (clone AFS98, host rat, BioLegend), Brilliant Violet 510 CD43 (clone 1G10, host mouse, BD Biosciences), and APC/cyanine7 F4/80 (clone BM8, host rat, BioLegend) for 45 minutes on ice. To assess bacterial phagocytosis in peritoneal exudate leukocytes, exudate cells were incubated with anti–mouse CD16/CD32 (clone 93, eBioscience; dilution 1:100, 20 minutes at 4°C in PBS containing 5% FCS staining solution) and then with Alexa Fluor 700–conjugated anti–mouse Ly6G (for neutrophils), APC/Cy7-conjugated anti–mouse F4/80, and PE-conjugated anti–mouse CD64 antibodies (monocyte-derived macrophages; 30 minutes at 4°C in staining solution). Cells were then fixed and permeabilized using BD Perm/Wash Buffer (BD Biosciences) following the manufacturer’s instructions and incubated with FITC-conjugated anti–*E*. *coli* antibody (catalog GTX13626, GenTex; 1:50 dilution, 30 minutes at 4°C in BD Perm/Wash Buffer).

We assessed efferocytosis in infectious exudates by incubating exudate leukocytes with anti–mouse CD16 and anti–mouse CD32 and then with APC- and Cy7-conjugated anti–mouse F4/80 and PE-conjugated anti–mouse CD64 antibodies (30 minutes at 4°C in staining solution). Cells were then fixed and permeabilized using BD Perm/Wash Buffer (BD Biosciences) and incubated with FITC-conjugated anti–mouse Ly6G antibody (30 minutes at 4°C, in BD Perm/Wash Buffer).

In select experiments, resolution indices were calculated as described previously (17, 18), where the time difference for neutrophil numbers to go from maximum to half maximum was determined as the R_i_. To assess the pro-resolving actions of the test products, these products were administered 12 hours after *E*. *coli* (10^5^ CFU) inoculation, and resolution indices were assessed.

#### Inflammatory arthritis.

Ten-week-old male C57BL/6 mice (Charles River Laboratories, the supplier fed the mice a lab diet containing essential fatty acids) were administered 9 μg Accell Control siRNA or Accell Mouse *GPR101* siRNA SmartPool (Dharmacon) via i.p. injection. After 24 hours, mice were i.p. administered 100 μl K/BxN serum (provided by Mauro Perretti and Dianne Cooper, Queen Mary University of London, London, United Kingdom) on days 0 and 2 to initiate inflammatory arthritis. Clinical scores were monitored daily using a 26-point arthritic scoring system (11). Mouse ankles, wrists, pads, and digits were inspected daily for swelling and redness. Blood, paws, and intestines were collected on day 8.

#### Mouse peripheral blood leukocyte phagocytosis.

Six- to eight-week-old male C57BL/6 mice (Charles River Laboratories; the supplier fed the mice a lab diet containing essential fatty acids) were i.p. administered 8 μg Accell Control siRNA or Accell Mouse *GPR101* siRNA SMARTpool (Dharmacon). After 3 days, blood was collected via cardiac puncture and incubated with either RvD5_n-3__DPA_ (10 nM) or vehicle (PBS containing 0.1% ethanol) for 20 minutes at 37°C, and then with 5 × 10^7^ CFU fluorescently labeled *E*. *coli* for 60 minutes at 37°C. At the end of the incubations, RBCs were lysed using a BD Pharmingen Red Blood Cell Lysis Kit following the manufacturer’s instructions. The cells were then fixed and incubated with anti-CD16/CD32 antibody (20 minutes at 4°C) followed by AF700-conjugated anti–mouse Ly6G (clone 1A8, BioLegend; for neutrophils) and BV-421–conjugated anti–mouse CD115 (clone AFS98, BioLegend; for monocytes) antibodies (30 minutes at 4°C). Cells were then washed and staining evaluated using a BD Fortessa II with BD FACSDiva software, and the results were analyzed using FlowJo software, version 10.4 (Tree Star).

### Blood collection from healthy volunteers

Venous peripheral blood was collected at the indicated intervals in sodium citrate (3.2%) from fasting volunteers who declared they had not taken NSAIDS for at least 14 days or consumed caffeine or alcohol for at least 24 hours or fatty fish for at least 48 hours.

### Human neutrophil responses

#### Human neutrophil chemotaxis.

Human peripheral blood neutrophils were isolated from healthy volunteers as detailed above. Cells were incubated with either an antibody against GPR101 or an isotype control for 15 minutes at room temperature and then with RvD5_n-3__DPA_ (0.1–10 nM) or vehicle control (PBS containing 0.01% ethanol) for 15 minutes at 37°C. Neutrophils were then transferred to the top chamber of a preloaded cell invasion and migration plate (CIM-Plate 16) on an xCELLigence DP system, and chemotaxis toward LTB_4_ (10 nM) was assessed in real time for 30 minutes.

#### Human neutrophil–endothelial cell interactions.

Human neutrophils, isolated as detailed above, were incubated with either an antibody against GPR101 or an isotype control for 15 minutes at room temperature and then with RvD5_n-3__DPA_ (0.1–10 nM) or vehicle control (PBS containing 0.01% ethanol) for 15 minutes at 37°C. Cells were then perfused over TNF-α–activated human umbilical endothelial cells (10 ng/mL, 4 hours) at 0.1 PA for 8 minutes, 6 random 10-second fields were recorded per condition, and neutrophil–endothelial cell interactions were quantified as previously described (4).

### Human macrophage responses

#### Human monocyte–derived macrophage efferocytosis.

PBMCs from healthy volunteers were purchased from the NHS Blood and Transplant Bank. PBMCs were isolated using Histopaque 1077 (MilliporeSigma) density centrifugation from blood cones. Macrophages were prepared using previously published protocols (31). Briefly, PBMCs were plated onto 10-cm tissue culture plates and incubated at 37°C for 30 minutes in PBS containing calcium and magnesium. Subsequently, cells were washed using PBS without calcium and magnesium, and adherent cells were incubated in RPMI 1640 containing 10% human serum and 20 ng/mL granulocyte macrophage–CSF (GM-CSF) for 7 days at 37°C in 5% CO_2_.

On day 7, monocyte-derived macrophages were then seeded onto 96-well plates at 4 × 10^4^ cells per well and transfected with 1 μM Accell Human Control siRNA or Accell Anti–human *GPR101* siRNA SMARTpool for 72 hours in serum-free Accell siRNA Delivery Medium (Dharmacon). Apoptotic target cells for efferocytosis were generated as follows: human promyelocytic HL-60 cells were seeded at 1 × 10^6^ cells/mL onto 35-mm plates, irradiated with UV-C light (254 nm) for 15 minutes, and incubated at 37°C for 2 hours. Apoptosis induction was verified by flow cytometry using the APC Annexin V Apoptosis Detection Kit with PI (BioLegend), in which annexin V–positive and annexin V/PI–double-positive cells were considered to be apoptotic. Apoptotic HL-60 cells were washed with PBS and labeled with 1 μM pHrodo Red Succinimidyl Ester (Invitrogen, Thermo Fisher Scientific) in PBS for 30 minutes at room temperature. Macrophages were stained with CellBrite Blue (Biotium) for 1 hour at 37°C to visualize cell membranes, washed with RPMI-1640, and incubated with RvD5_n-3__DPA_ (0.001–10 nM) or vehicle (RPMI-1640 containing 0.1% ethanol) for 15 minutes at 37°C. Apoptotic pHrodo Red–labeled HL-60 cells were added directly to the macrophages at a 3:1 ratio (apoptotic HL-60/macrophage), and the increase in pHrodo Red signal over time (representing apoptotic cell efferocytosis by macrophages) was quantified using a Zeiss Celldiscoverer 7 high-content imaging system.

#### Human monocyte–derived macrophage phagocytosis.

Monocyte-derived macrophages were seeded onto 96-well plates at 4 × 10^4^ cells per well and transfected with 1 μM Accell Human Control siRNA or Accell Anti–human *GPR101* siRNA SMARTpool for 72 hours in serum-free Accell siRNA Delivery Medium (Dharmacon). Macrophages were stained with Hoechst 33342 (Thermo Fisher Scientific) for 1 hour at 37°C to visualize cell nuclei, washed with RPMI-1640, and incubated with RvD5_n-3__DPA_ (0.001–10 nM) or vehicle (RPMI-1640 containing 0.1% ethanol) for 15 minutes at 37°C. pHrodo Green–labeled *S. aureus* bioparticle conjugates (Invitrogen, Thermo Fisher Scientific) were opsonized by incubation in PBS containing 20% (v/v) human serum. Opsonized pHrodo Green–labeled *S*. *aureus* bioparticles were directly added to the macrophages at a final concentration of 5 μg per well, and the increase in pHrodo Green signal over time (representing phagocytosis of bacterial particles by macrophages) was quantified using a Zeiss Celldiscoverer 7 high-content imaging system.

#### cAMP measurements.

Human monocyte–derived macrophages were seeded onto 12-well plates at 5 × 10^5^ cells per well and then transfected with 1 μM Accell Human Control siRNA or Accell Anti–human *GPR101* siRNA SMARTpool for 72 hours in serum-free Accell siRNA Delivery Medium (Dharmacon). Cells were incubated with RvD5_n-3__DPA_ (1 nM) or vehicle (PBS containing 0.01% ethanol) for 2 minutes, after which incubations were quenched with 5% Triton X-100, cells were homogenized, and cAMP levels were determined by ELISA following the manufacturer’s instruction (Elite cAMP ELISA Assay kit, eEnzyme).

#### Monocyte-derived macrophage l-kynurenine.

Macrophages were prepared as detailed above and then seeded onto 12-well plates at 2.5 × 10^5^ cells per well and kept overnight in RPMI 1640 containing 10% FBS. Cells were then incubated with 10 nM RvD5_n-3__DPA_ or vehicle. Two hours later, cells were collected using methanol containing deuterium-labeled choline and subjected to LC-MS/MS analysis as detailed previously (11) to determine l-kynurenine concentrations.

### Statistical analysis

We performed all statistical analyses and data derivation using GraphPad Prism 8 (GraphPad Software) and Microsoft Excel software. Results presented in the figures are expressed as the mean ± SEM. Statistical differences between groups were determined as described in the figure legends. Briefly, Gaussian distribution of the data was tested using the Shapiro-Wilk normality test. For data following a normal distribution, statistical differences were tested using 1-way or 2-way ANOVA with a Holm-Sidak or Tukey’s post hoc multiple comparisons tests. For data not following a normal distribution, a Kruskal-Wallis test with Dunn’s post hoc multiple comparisons test was used. Sample sizes for each experiment were determined on the basis of the variability observed in prior experiments.

### Study approval

Animal experiments were approved by the United Kingdom Home Office, London, United Kingdom (P998AB295) and adhered to the Guidance on the Operation of Animals, Scientific Procedures Act and to Laboratory Animal Science Association Guidelines (*Guiding Principles on Good Practice for Animal Welfare and Ethical Review Bodies*, Royal Society for the Prevention of Cruelty to Animals [RSPCA], 2015). Studies involving human primary cells were approved by the Queen Mary Research Ethics Committee, London, United Kingdom (QMREC 2014:61). All volunteers provided informed consent before blood donation. Healthy volunteers who donated blood gave written consent in accordance with a Queen Mary Research Ethics Committee (QMREC 2014:61) and Declaration of Helsinki principles.

## Author contributions

JD conceived the overall research plan. MBF, DSK, AS, JS, KP, FP, and JD conducted the experiments and analyzed results. MBF and JD wrote the manuscript. All authors contributed to manuscript preparation. The order of the co–second authors was assigned on the basis of both experimental and intellectual contributions.

## Supplementary Material

Supplemental data

## Version 1 12/03/2019

Electronic publication

## Version 2 01/02/2020

Print issue publication

## Version 3 12/12/2022

Legend text for Figure 2C edited for clarity
