# Supplementary material for: GPR101 mediates the pro-resolving actions of RvD5n-3 DPA in arthritis and infections
Source: J Clin Invest. 2019 Dec 3;130(1):359–73. doi: 10.1172/JCI131609 (PMC6934227; doi:10.1172/JCI131609)
Supplement: Supplemental data [file jci-130-131609-s009.pdf]

GPR101 mediates the pro-resolving actions of RvD5$_{n\text{-}3\ DPA}$ in arthritis and infections

Magdalena B. Flak[1], Duco S. Koenis[1*], Agua Sobrino[1*], James Smith[1], Kimberly Pistorius[1], Francesco Palmas[1], Jesmond Dalli[1, 2 +].

*share second authorship

[1] William Harvey Research Institute, Barts and The London School of Medicine and Dentistry, Queen Mary University of London, Charterhouse Square, London, EC1M 6BQ, UK.

[2] Centre for Inflammation and Therapeutic Innovation, Queen Mary University of London, London, UK.

[+]Corresponding author: Dr Jesmond Dalli Ph.D, Lipid Mediator Unit, William Harvey Research Institute, John Vane Science Centre, Charterhouse Square, London EC1M 6BQ. E-mail: j.dalli@qmul.ac.uk, Tel: +44 (0) 207 882 8263

Keywords:

lipid mediators, resolvins, specialized pro-resolving mediators, rheumatoid arthritis, G-protein coupled receptor, omega-3

Supplemental Figures

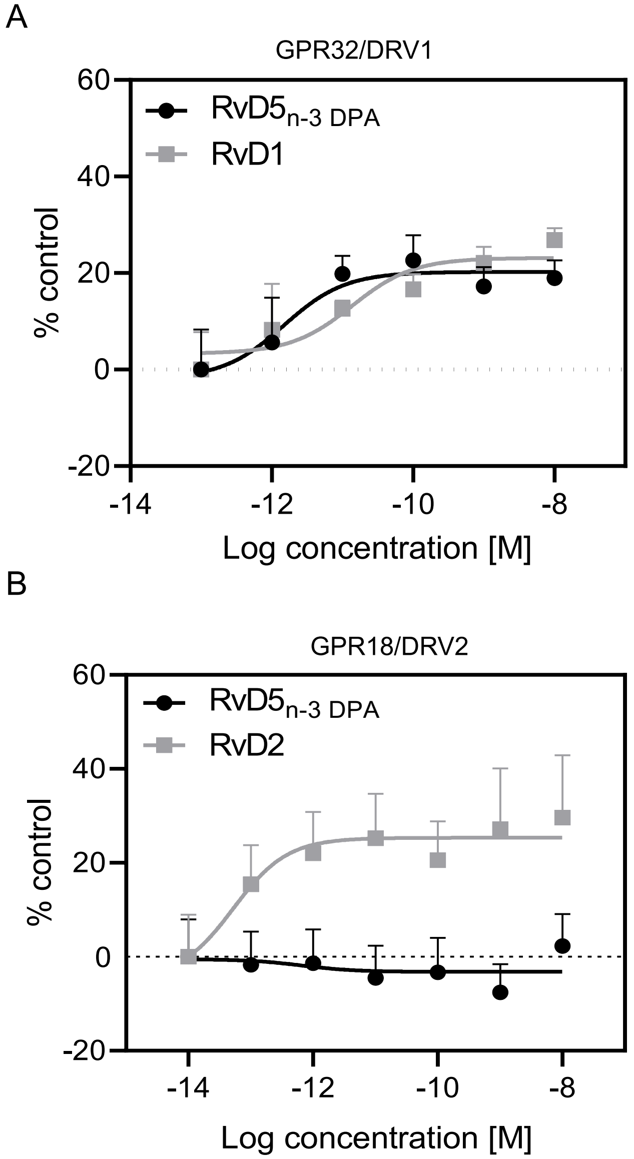

Supplemental Figure 1: Interaction of RvD5$_{n\text{-}3\ DPA}$ with GPR32/DRV1 and GPR18/DRV2. (A) CHO cells expressing GPR32 coupled with the β-arrestin luminescent reporter system were incubated with the indicated concentrations of RvD1 (EC_{50} ~1.4×10^{-11} M), RvD5$_{n\text{-}3\ DPA}$ (EC_{50} ~1.5×10^{-12} M), or vehicle (Cell Plating Reagent containing 0.01% ethanol) and receptor activation was measured as an increase in luminescence signal. (B) CHO cells expressing

GPR18 coupled with the β-arrestin luminescent reporter system were incubated with the indicated concentrations of RvD2 (EC_{50} ~5.3 x 10^{-14} M), $RvD5_{n\text{-}3\ DPA}$ (no response), or vehicle (Cell Plating Reagent containing 0.01% ethanol) and receptor activation was measured as an increase in luminescence signal. Results are shown as mean ± SEM (n = 3 in two independent experiments).

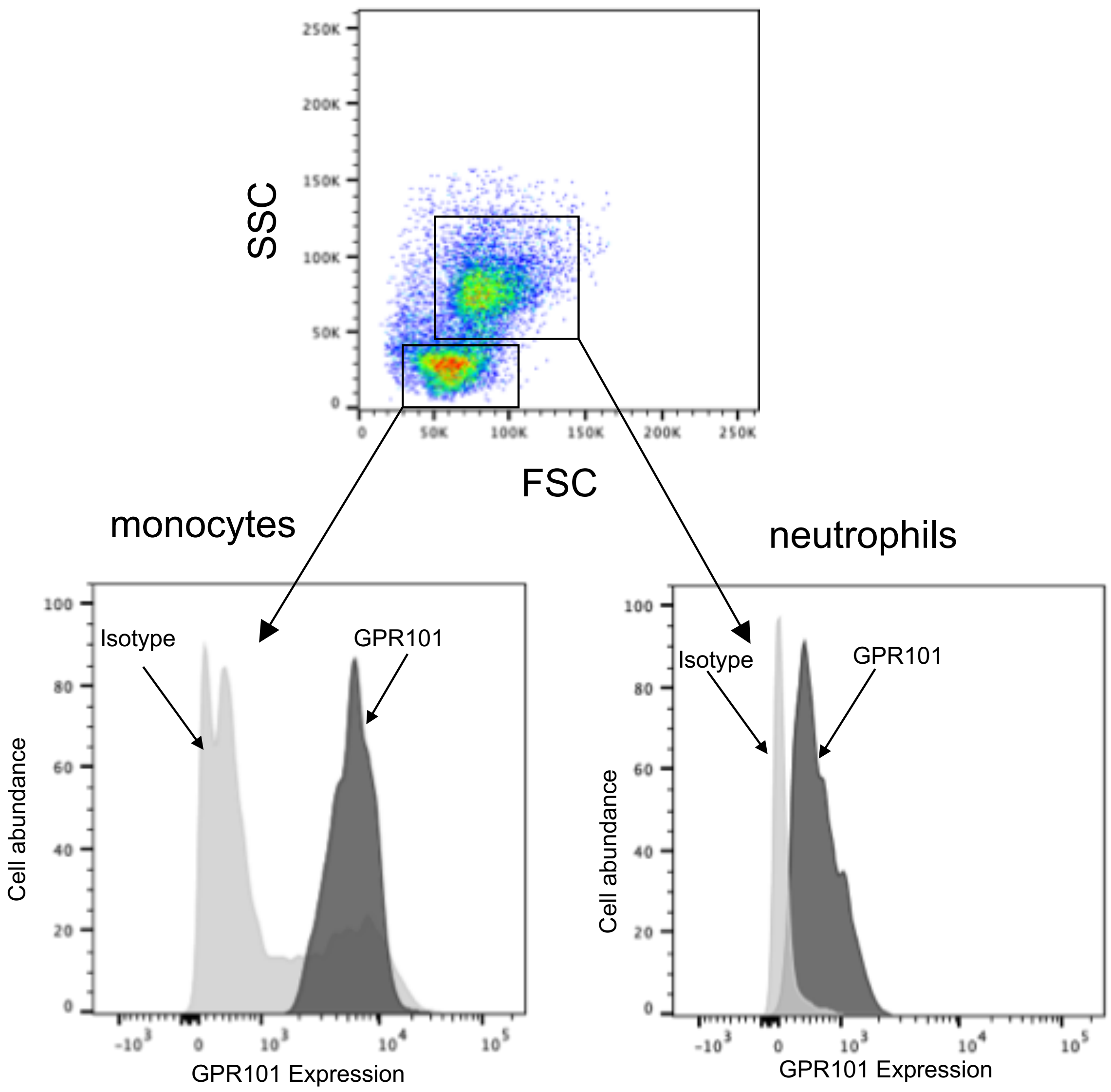

Supplemental Figure 2: Expression of GPR101 in mouse leukocytes. Peripheral blood was collected and the expression of GPR101 was determined in neutrophils and monocytes using flow cytometry. Results are representative of n = 4 mice per group from two distinct experiments.

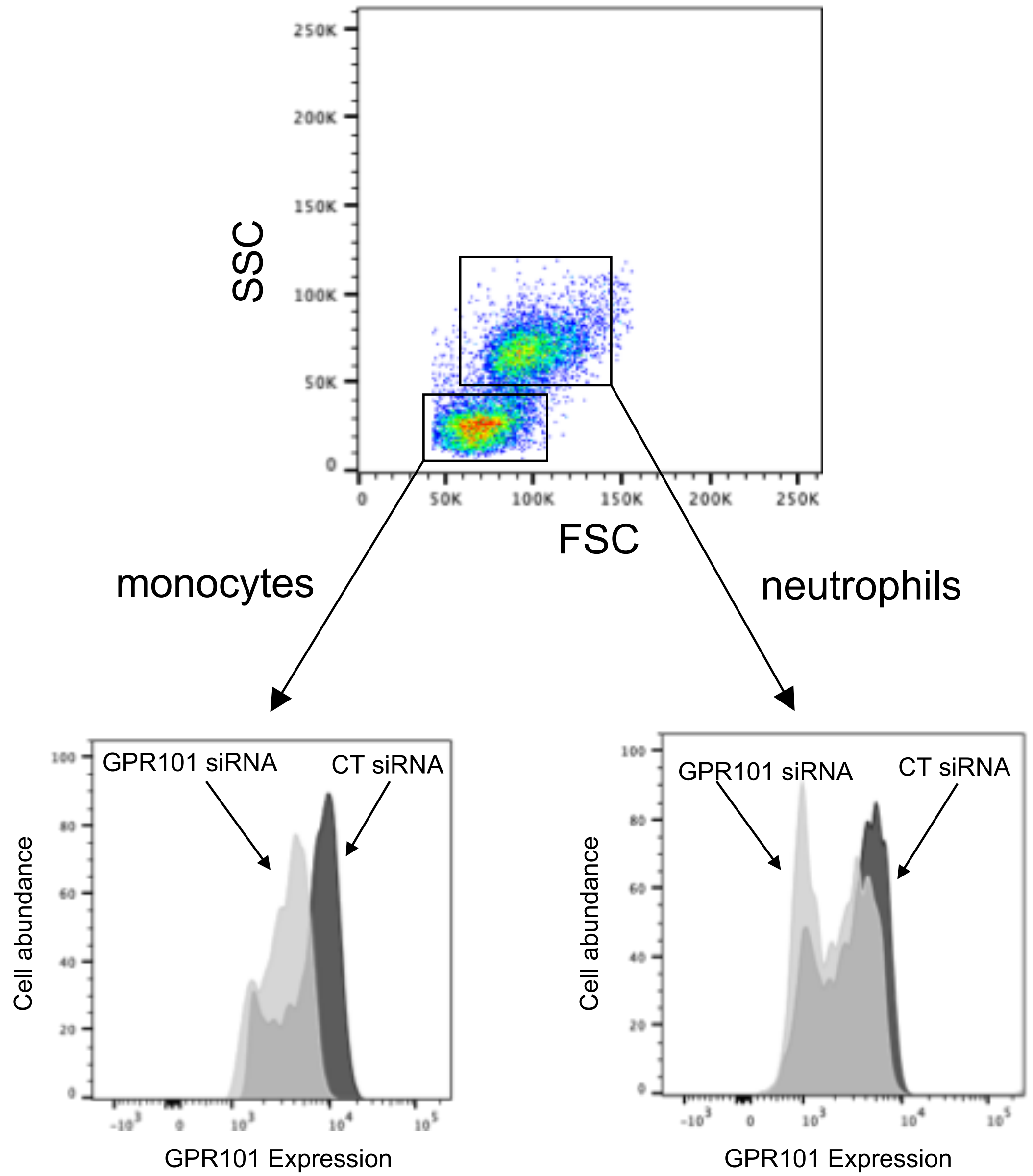

Supplemental Figure 3: Administration of siRNA targeting GPR101 reduces receptor expression on mouse circulating neutrophils and monocytes. Mice were administered 9 µg of siRNA to mouse GPR101 or a scrambled control sequence (CT siRNA). After 72 h blood was collected and the expression of GPR101 was determined on neutrophils and monocytes. Results are representative of n = 4 mice per group from two distinct experiments.

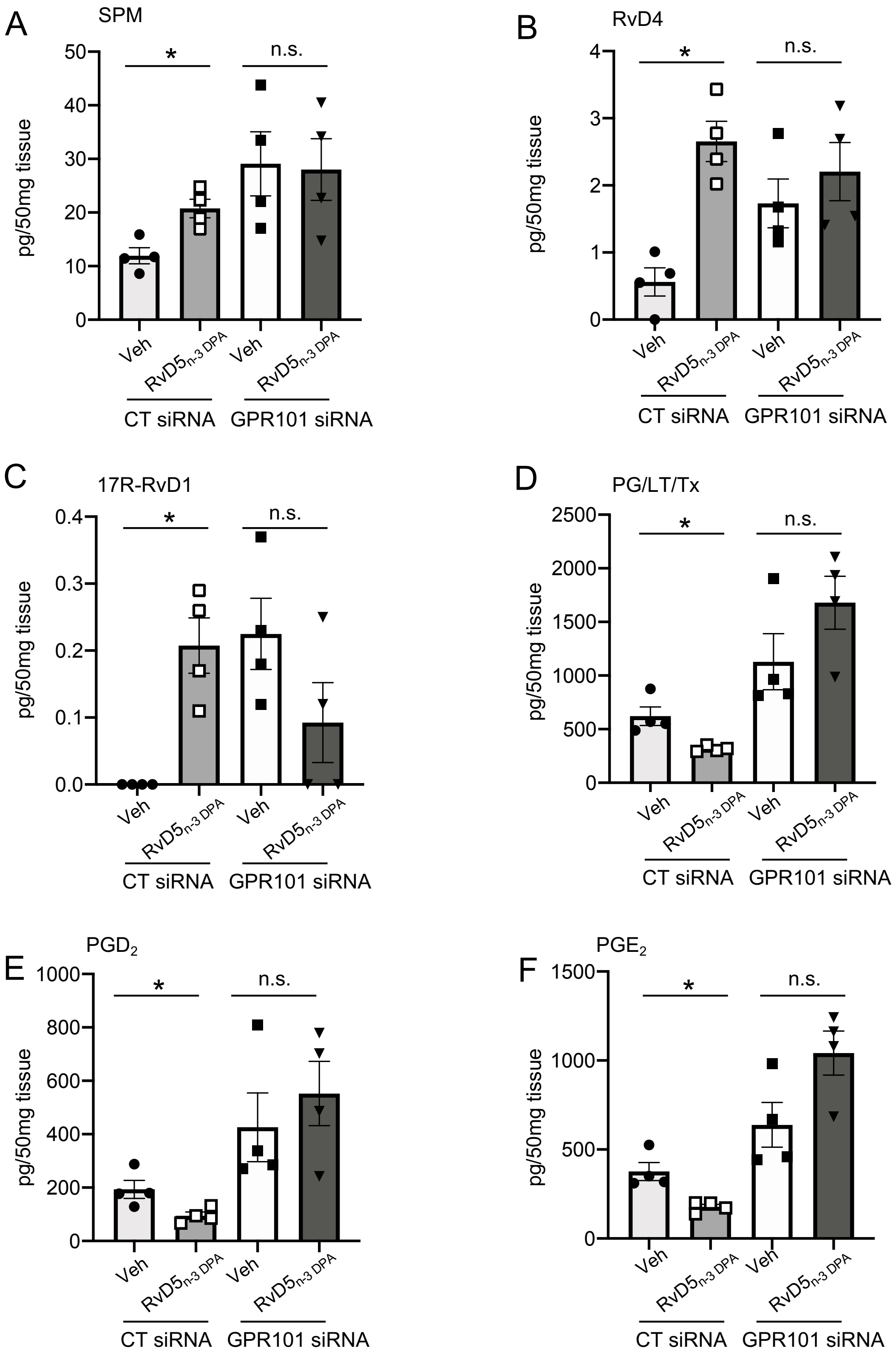
A
SPM
B
RvD4
C
17R-RvD1
D
PG/LT/Tx
E
PGD2
F
PGE2
*
n.s.
pg/50mg tissue
Veh
RvD5n-3 DPA
CT siRNA
GPR101 siRNA

Supplemental Figure 4: Reduction of GPR101 expression limits the ability of $RvD5_{n\text{-}3\ DPA}$ to regulate intestinal eicosanoid and SPM concentrations. Mice were administered 9 µg of siRNA to mouse GPR101 or a scrambled control sequence (CT siRNA). After 24 h and 72 h mice were administered arthritogenic serum. Mice were then treated with $RvD5_{n\text{-}3\ DPA}$ (150ng/mouse) or vehicle (72h and 96h after siRNA administration), small intestines were collected on day 7 post serum administration and lipid mediator profiles determined using LC-MS/MS based lipid mediator profiling. Concentrations of (A) pro-resolving mediators (sum of DHA, n-3 DPA, EPA and AA derived specialized pro-resolving mediators- SPM), (B) RvD4, (C)17R-RvD1, (D) sum of prostaglandins (PG), Leukotrienes (LT) and Thromboxane (Tx), (E) PGD_2, (F) PGE_2. Results are representative of n = 4 mice per group. * $P < 0.05$ *versus* vehicle group using Kruskal-Wallis test with Dunn's post hoc multiple comparisons test.

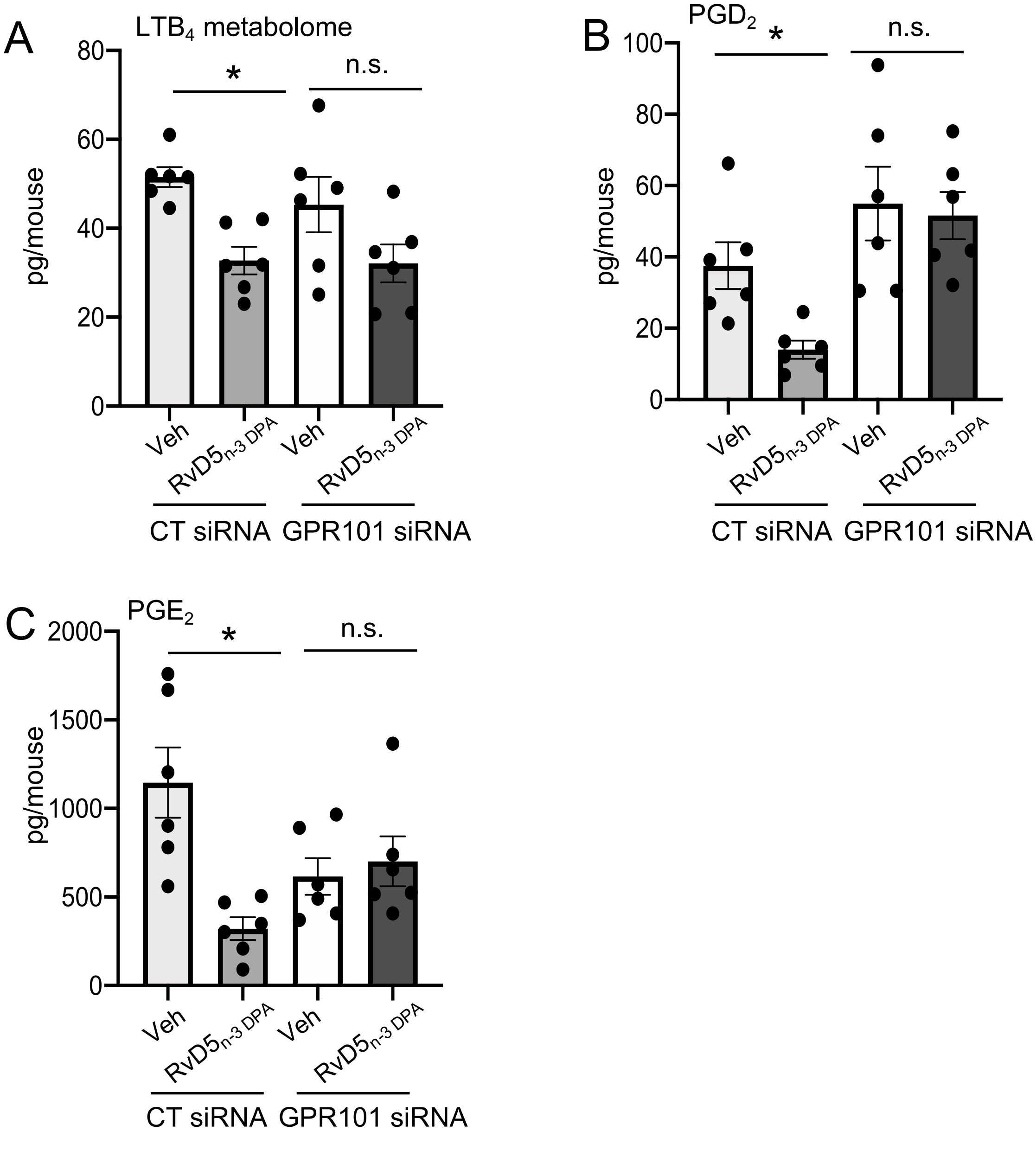

Supplemental Figure 5: Knockdown of GPR101 limits the ability of RvD5$_{n\text{-}3\ DPA}$ to regulate exudate prostaglandin and leukotriene concentrations. Mice were administered 9 µg of siRNA to mouse GPR101 or a scrambled control sequence (CT siRNA). After 72 h they were administered RvD5$_{n\text{-}3\ DPA}$ (100ng/mouse) or vehicle control (PBS containing 0.1 % ethanol) then inoculated via intraperitoneal injection with 10^5 c.f.u. *E. coli*. After 14h exudates were collected

and concentrations of (A) the Leukotriene B_4 metabolome (LTB_4, 5S, 12S-diHETE and 20-OH-LTB_4), (B) PGD_2, (C) PGE_2 were determined using LC-MS/MS based lipid mediator profiling. Results are representative of n = 6 mice per group from two distinct experiments. * $P < 0.05$ *versus* vehicle group using Kruskal-Wallis test with Dunn's post hoc multiple comparisons test.

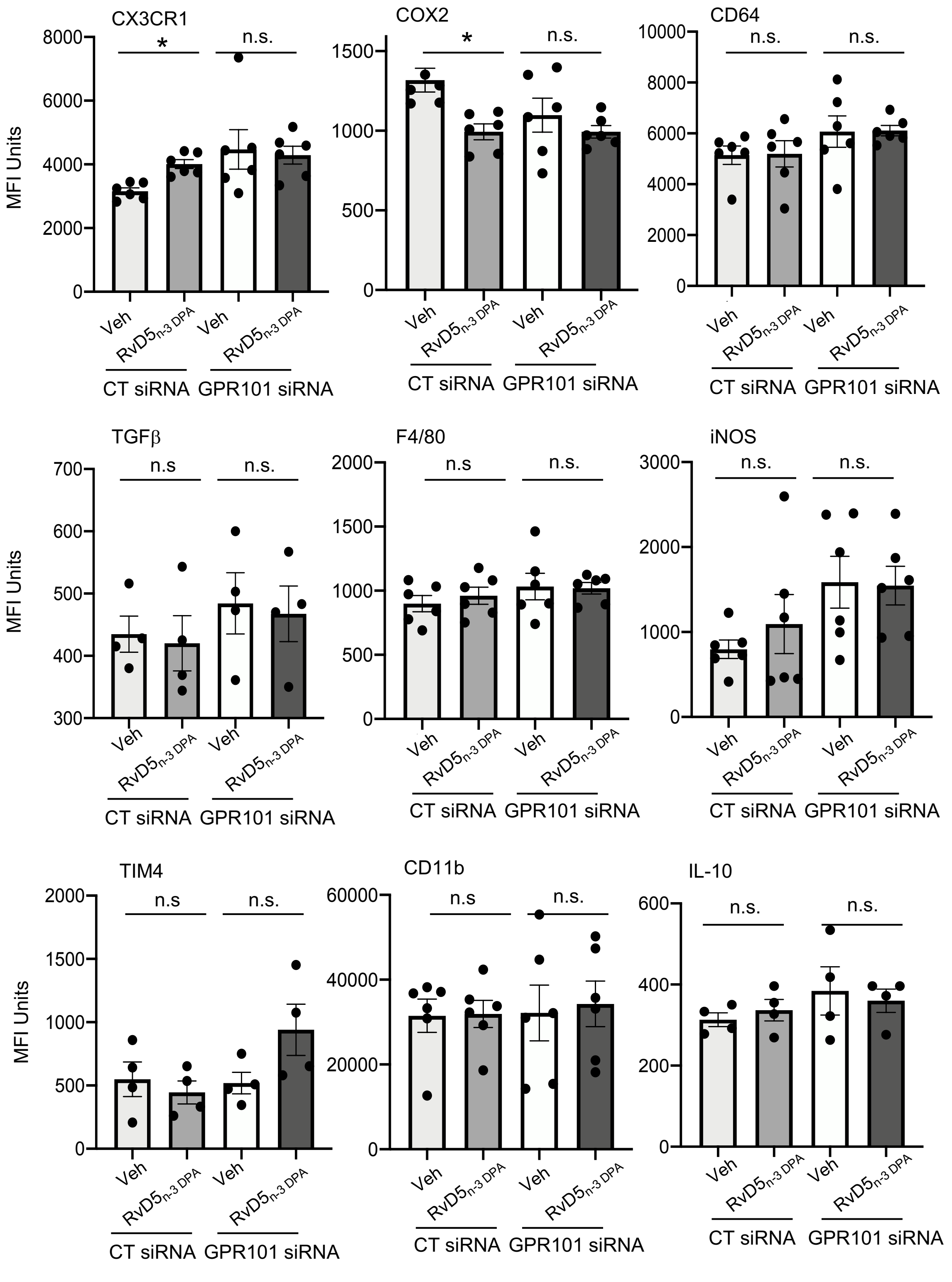

Supplemental Figure 6: Knockdown of GPR101 limits the ability of RvD5$_{n\text{-}3\ DPA}$ to regulate exudate monocyte-derived macrophage phenotype during infectious inflammation. Mice

were administered 9 µg of siRNA to mouse GPR101 or a scrambled control sequence (CT siRNA) after 72 h they were administered RvD5$_{n\text{-}3\ DPA}$ (100ng/mouse) or vehicle control (PBS containing 0.1 % ethanol) then inoculated with 10^5 c.f.u. *E. coli*. After 14h exudates were collected and the expression of macrophage lineage markers was determined using fluorescently labelled antibodies and flow cytometry. Results are representative of n = 4-6 mice per group from two distinct experiments. *P < 0.05 *versus* vehicle group using Kruskal-Wallis test with Dunn's post hoc multiple comparisons test.

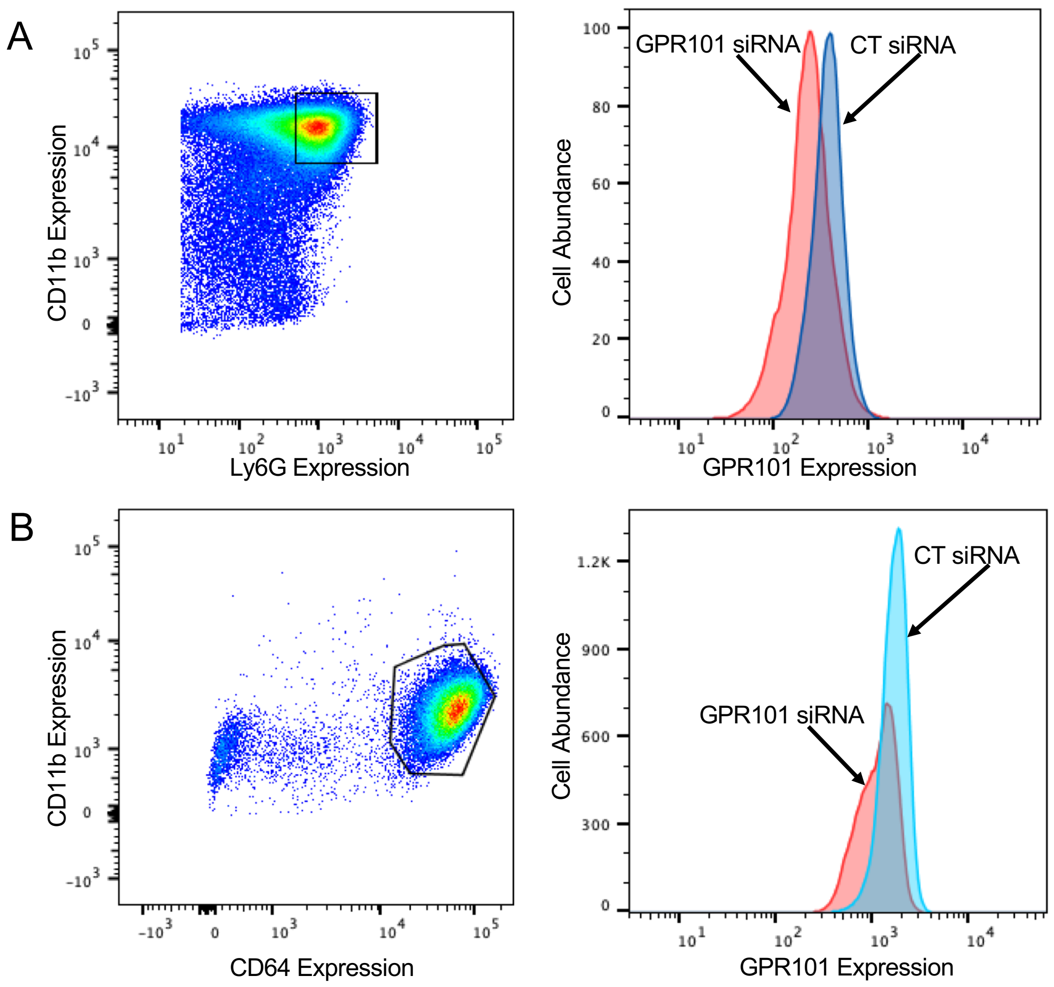

Supplemental Figure 7: Administration of siRNA targeting GPR101 reduces receptor expression on mouse peritoneal neutrophils and macrophages during *E. coli* infections. Mice were administered 9 µg of siRNA to mouse GPR101 or a scrambled control sequence (CT siRNA). After 72 h they were challenged with *E. coli* (10^5 c.f.u./mouse) and exudates collected after 4h. GPR101 Expression was determined on (A) neutrophils and (B) macrophages using flow cytometry and fluorescently labelled antibodies. Results are representative of n = 4 mice per group.
